# Baicalin inhibits biofilm formation, attenuates the quorum sensing-controlled virulence and enhances *Pseudomonas aeruginosa* clearance in a mouse peritoneal implant infection model

**DOI:** 10.1371/journal.pone.0176883

**Published:** 2017-04-28

**Authors:** Jing Luo, Biying Dong, Ke Wang, Shuangqi Cai, Tangjuan Liu, Xiaojing Cheng, Danqing Lei, Yanling Chen, Yanan Li, Jinliang Kong, Yiqiang Chen

**Affiliations:** 1 Department of Respiratory Disease, First Affiliated Hospital of Guangxi Medical University, Qingxiu, Nanning, Guangxi, People’s Republic of China; 2 Life Sciences Institute of Guangxi Medical University, Qingxiu, Nanning, Guangxi, People’s Republic of China; Purdue University, UNITED STATES

## Abstract

The quorum sensing (QS) circuit plays a role in the precise regulation of genes controlling virulence factors and biofilm formation in *Pseudomonas aeruginosa*. QS-controlled biofilm formation by *Pseudomonas aeruginosa* in clinical settings has remained controversial due to emerging drug resistance; therefore, screening diverse compounds for anti-biofilm or anti-QS activities is important. This study demonstrates the ability of sub-minimum inhibitory concentrations (sub-MICs) of baicalin, an active natural compound extracted from the traditional Chinese medicinal *Scutellaria baicalensis*, to inhibit the formation of *Pseudomonas aeruginosa* biofilms and enhance the bactericidal effects of various conventional antibiotics *in vitro*. In addition, baicalin exerted dose-dependent inhibitory effects on virulence phenotypes (LasA protease, LasB elastase, pyocyanin, rhamnolipid, motilities and exotoxin A) regulated by QS in *Pseudomonas aeruginosa*. Moreover, the expression levels of QS-regulatory genes, including *lasI*, *lasR*, *rhlI*, *rhlR*, *pqsR* and *pqsA*, were repressed after sub-MIC baicalin treatment, resulting in significant decreases in the QS signaling molecules 3-oxo-C12-HSL and C4-HSL, confirming the ability of baicalin-mediated QS inhibition to alter gene and protein expression. *In vivo* experiments indicated that baicalin treatment reduces *Pseudomonas aeruginosa* pathogenicity in *Caenorhabditis elegans*. Greater worm survival in the baicalin-treated group manifested as an increase in the LT_50_ from 24 to 96 h. In a mouse peritoneal implant infection model, baicalin treatment enhanced the clearance of *Pseudomonas aeruginosa* from the implants of mice infected with *Pseudomonas aeruginosa* compared with the control group. Moreover, the combination of baicalin and antibiotics significantly reduced the numbers of colony-forming units in the implants to a significantly greater degree than antibiotic treatment alone. Pathological and histological analyses revealed mitigation of the inflammatory response and reduced cell infiltration in the peritoneal tissue surrounding the implants after baicalin treatment. Measurement of the cytokine levels in the peritoneal lavage fluid of mice in the baicalin treatment group revealed a decrease in IL-4, an increase in interferon γ (IFN-γ), and a reversed IFN-γ/IL-4 ratio compared with the control group, indicating that baicalin treatment activated the Th1-induced immune response to expedite bacterial load clearance. Based on these results, baicalin might be a potent QS inhibitor and anti-biofilm agent for combating *Pseudomonas aeruginosa* biofilm-related infections.

## Introduction

*Pseudomonas aeruginosa*, a gram-negative opportunistic pathogen, is one of the major causes of nosocomial infections. In immunocompromised patients with cystic fibrosis, burn wounds and indwelling devices, *P*. *aeruginosa* infections are particularly common and result in high morbidity and mortality [1,2]. Like many other pathogens, *P*. *aeruginosa* forms biofilms on damaged tissue or medical implants in addition to living as free planktonic cells within the host [3]. Notably, biofilms comprise more than 80% of all microbial infections associated with catheters, foreign-body implants, urinary tract infections, dental plaque, and gingivitis and commonly produce chronic and persistent infections [4–6]. Compared with their free planktonic cell counterparts, bacterial communities embedded in biofilms exhibit distinctive growth, gene expression and cell-cell communication phenotypes [7]. When protected by highly hydrated extracellular polymeric substances, *P*. *aeruginosa* can survive host immune attacks and becomes up to 1000-fold more resistant to conventional antibiotics; thus, biofilm-associated infections are often very difficult to treat in the clinic [8]. Therefore, combating *P*. *aeruginosa* biofilm infections requires novel targets.

To facilitate the establishment of infection, *P*. *aeruginosa* cells in biofilms coordinate their cell density-dependent gene expression by excreting small signaling molecules. When the bacteria reach a population density threshold, these small signaling molecules bind to a specific receptor, thereby activating the quorum sensing (QS) system [9]. *P*. *aeruginosa* employs two well-defined acyl homoserine lactone (AHL)-based QS systems, the *lasI/lasR* and *rhlI/rhlR* systems; these systems are arranged in a hierarchical manner, but the *lasI/lasR* system is dominant and positively upregulates the activity of the *rhlI/rhlR* system [10]. In the *lasI/lasR* system, *lasI* is responsible for synthesizing N-(3-oxo-dodecanoyl)-L-homoserine lactone (3-oxo-C_12_-HSL) [11]. When the concentration of 3-oxo-C_12_-HSL exceeds a certain threshold, this molecule binds to the cytoplasmic receptor *LasR* and activates the expression of genes that produce virulence factors, such as LasA proteases, LasB elastases and exotoxin A [12]. As a homolog of *LasI*, *rhlI* is regulated by *LasR-*3*-*oxo-C_12_-HSL. The *rhl* system functions via its signaling molecule N-butanoyl-L-homoserine lactone (C_4_-HSL) [11]; similar to 3-oxo-C_12_-HSL, C_4_-HSL binds to the receptor *RhlR* and activates certain target genes, including the genes responsible for the production of pyocyanin, rhamnolipids and siderophores [13]. There is also a third, intermediate, non-AHL-based system known as *Pseudomonas* quinolone signal (PQS), which utilizes 2-heptyl-3-hydroxy-4-quinolone as its signaling molecule. PQS is regulated by the *lasI/lasR* system and enhances the expression of *rhlI* and *rhlR* [14,15]. In *P*. *aeruginosa*, after the QS system is blocked either by the mutation of QS-regulated genes or by the administration of QS-inhibiting agents, biofilm formation, virulence, tissue damage and the mortality rate are attenuated [16–19]. Moreover, QS deficiency increases bacterial susceptibility to tobramycin. Combination treatment with a QS inhibitor and tobramycin exerts synergistic effects on biofilm killing *in vitro* and *in vivo* [16,20]. Therefore, the disruption of QS in *P*. *aeruginosa* has been proposed as a promising anti-biofilm and anti-infection strategy to combat the emergence of antibiotic-resistant phenotypes, which has resulted in a search for new therapeutic alternatives [11,16,18].

Plant-derived compounds have been widely used for centuries to combat microbial infections because they are considered safe for human consumption. Baicalin (5,6,7-trihydroxyflavone), one of the major flavonoid monomers purified from the roots of *Scutellaria baicalensis*, has been described as an herb in the Chinese Pharmacopoeia. Numerous traditional Chinese medicine (TCM) formulae containing baicalin are widely used clinically for treating fever, bronchitis and upper respiratory tract infections [21–23]. Additionally, baicalin exerts antifungal activity against *Candida albicans* [24], antiviral activity against enteroviruses [25] and antibacterial activity against methicillin-resistant *Staphylococcus aureus* [26], *Helicobacter pylori* [27] and *Escherichia coli* [28], among others. According to Brackman et al. [16], the hydrate of baicalin exerts a synergetic effect with tobramycin to kill biofilm-associated *P*. *aeruginosa* cells in a colony count assay; baicalin also inhibited AHL-based QS-regulated gene expression in *Burkholderia cenocepacia* [29]. However, no specific evidence has demonstrated the effects of baicalin on morphological alterations to *P*. *aeruginosa* biofilms or combinatorial effects with other classes of antibiotics, such as fluoroquinolones, cephalosporins and even aminoglycosides other than tobramycin. Further, evidence is still lacking regarding the effects of baicalin on QS-controlled virulence and gene expression in *P*. *aeruginosa*.

In the present study, we first sought to determine whether baicalin effectively prevents early and mature *P*. *aeruginosa* biofilm formation and to assess the combined efficacy of baicalin with representatives of three completely different classes of antibiotics (levofloxacin, tobramycin and ceftazidime) that are widely employed clinically to cure *P*. *aeruginosa* infection by killing biofilm-associated bacteria. Furthermore, we assayed the influence of baicalin on *P*. *aeruginosa* QS-controlled virulence factor production. The mechanisms underlying these interventions were investigated by detecting the expression levels of QS-regulated genes and AHL-based QS molecules in *P*. *aeruginosa*. Additionally, treatment with baicalin is a potential strategy to attenuate *P*. *aeruginosa* pathogenesis in *P*. *aeruginosa*-infected *C*. *elegans* and to enhance bacterial clearance in a mouse model of peritoneal implant infection. Our work reveals the importance of baicalin as a potential anti-biofilm agent and QS inhibitor for treating *Pseudomonas aeruginosa* infection.

## Materials and methods

### Bacterial strains and reagents

This study used the wild-type sequenced *P*. *aeruginosa* strain PAO1, which is commonly used in current research investigating *P*. *aeruginosa* biofilms. *P*. *aeruginosa* PAO1 and its *lasI-rhlI*-deficient mutant (*ΔlasI-ΔrhlI*) were both generously donated by Yang Liang, PhD, at Nanyang Technological University, Singapore. The quality control strain *P*. *aeruginosa* ATCC27853 was purchased from the American Type Culture Collection. The strains were maintained at -80°C in Luria–Bertani (LB, Sigma-Aldrich, St. Louis, MO, USA) broth containing 25% glycerol. Prior to each experiment, one loop of bacterial stock solution was resuscitated and streaked onto an LB agar plate. A single colony was picked and subcultured in LB broth or Mueller-Hinton (M-H, Landbridge Co., Beijing, China) broth for 16–18 h at 37°C and 200 rpm continuous shaking to determine the minimum inhibitory concentration (MIC) and minimum bactericidal concentration (MBC). Following incubation, the overnight culture was diluted with the same medium to the appropriate bacterial cell density for subsequent experiments.

*Caenorhabditis elegans* N2 and *E*. *coli* OP50 were both donated by Professor Qichun Huang, head of the Department of Clinical Medicine, Affiliated Tumor Hospital of Guangxi Medical University. The nematode was propagated under standard conditions, synchronized via hypochlorite bleaching, and cultured on nematode growth medium (NGM, US Biological, Salem, MA, USA) at 20°C using *E*. *coli* OP50 as the standard food source.

For *in vitro* studies, baicalin was purchased from Sigma-Aldrich (St. Louis, MO, USA) as a standard dry powder with a purity of 95% that was confirmed by high-performance liquid chromatography. Baicalin was freshly dissolved in dimethyl sulfoxide (DMSO, Amresco, Solon, OH, USA) and then sterilized by passage through a 0.22-μm syringe filter (Sigma-Aldrich, Merck Millipore, Darmstadt, Germany). For *in vivo* experiments, baicalin was dissolved in phosphate-buffered saline (PBS, Solarbio, Beijing, China), and sodium hydroxide was added to obtain a final pH of 7.4 to facilitate dissolution. Levofloxacin (LEV), amikacin (AMK) and ceftazidime (CAZ) were all USP grade and purchased from Sigma-Aldrich (St. Louis, MO, USA). Pure stock solutions of antibiotics were prepared in Milli-Q water at 10 mg/mL, sterilized by filtration, and stored at 4°C until use.

### MIC and MBC determination and growth curve assay

A broth-microdilution method from the Clinical and Laboratory Standards Institute standards [30] was employed to assess the MICs and MBCs of baicalin and antibiotics. Briefly, Two-fold serial dilutions of baicalin and antibiotics were prepared in M-H broth (pH 7.2–7.4), at a volume of 100 μL per well in 96-well U-bottomed polystyrene microtiter plates (Corning/Costar, NY, USA). Each well was inoculated with 100 μL of the standardized *P*. *aeruginosa* PAO1 inoculum, yielding a final bacterial concentration of approximately 1×10^5^ colony forming units (CFUs)/mL. The final tested concentrations of antimicrobial agents ranged from 2–1,024 μg/mL for baicalin, 0.125–16 μg/mL for LEV and 0.25–32 μg/mL for AMK and CAZ. The MIC was defined as the lowest concentration of the tested agent that resulted in the complete inhibition of visible growth in M-H broth. The MBC was defined as the lowest concentration of the tested agent that killed 99.9% of the test bacteria following plating onto M-H agar plates. The standard strain *P*. *aeruginosa* ATCC27853 was used for quality control to ensure the accuracy of the susceptibility results.

To construct growth curves, overnight cultures of *P*. *aeruginosa* PAO1 inocula were diluted in 100 mL of fresh LB broth to achieve a cell suspension optical density at 600 nm (OD_600_) of 0.05. The suspension was supplemented with sub-MICs of baicalin and incubated at 37°C under continuous agitation (200 rpm). At the time points indicated, 1-mL samples were obtained, and the turbidity was monitored as the OD_600_ using a spectrophotometer (Multiskan MK3, Thermo Fisher Scientific, Waltham, MA, USA). Corresponding data were continuously recorded to generate a growth curve.

### Biofilm assessment

#### Biofilm inhibition assay

To detect the inhibitory effects of baicalin on biofilm formation by *P*. *aeruginosa* PAO1, individual wells of sterile 96-well flat-bottomed polystyrene microtiter plates (Corning/Costar, NY, USA) were seeded with 100 μL of bacterial suspension prepared at approximately 1×10^7^ CFU/mL in the presence of sub-MICs of baicalin as indicated. The plates were covered and incubated statically at 37°C for the indicated periods in order to facilitate cell attachment and biofilm formation. Then, non-adherent cells in the supernatants were removed by pipetting, and the remaining biofilms were gently rinsed three times with sterile PBS. Biofilm biomass was assessed by performing a crystal violet assay [31], and viable bacteria in the biofilm were evaluated by obtaining colony counts as described below.

#### Assessment of biofilm dispersion and combination treatment with antibiotics

To evaluate pre-existing biofilm disassembly and the efficacy of combination treatments, prepared bacterial cultures at approximately 1×10^7^ CFU/mL were added to 96-well plates at 100 μL/well and incubated at 37°C for 24 h or 96 h to obtain premature and mature *P*. *aeruginosa* PAO1 biofilms, respectively. Subsequently, supernatants containing non-adherent cells were removed, and biofilms were gently rinsed three times with sterile PBS. Existing biofilms were incubated at 37°C in LB supplemented with the tested agents (baicalin alone, each antibiotic alone or baicalin + antibiotics) for the indicated periods of time. Baicalin was used at sub-MICs that did not affect bacterial growth, whereas antibiotics were used at MBCs to explore their bactericidal effect on sessile cells in biofilms alone and in combination with baicalin. Biofilm biomass and bacterial counts in biofilms were evaluated as described below.

#### Biofilm mass evaluation and bacterial colony count assay

A crystal violet staining protocol was used to quantitate biofilm mass after treatment [31]. After various incubation periods, as indicated, the culture medium was carefully pipetted from individual wells of a 96-well plate, and the plate was gently washed three times with PBS to remove any loosely attached bacterial cells. The remaining biofilms attached to the wells were then fixed with 150 μL of absolute methanol for 15 min and air-dried at room temperature for 10 min. Next, 100 μL of 1% (w/v) crystal violet was added and allowed to stain the samples for 20 min. Excess crystal violet was removed by rinsing the wells three times with sterile distilled water. The crystal violet-stained materials were then dissolved in absolute ethanol, and the amount of stained biomass was indirectly measured spectrometrically (at OD_595_). A bacterial colony count assay was performed to evaluate the biofilm bacterial burden after treatment. Briefly, 0.1% Triton X-100 (Sangon Biotech, Shanghai, China) was added to each PBS-rinsed well, and the plate was sonicated in an ice bath for 5 min using a Bransonic 220 instrument (80 W, 42 kHz) to detach the remaining biofilms. Serial dilutions from each well were plated onto LB agar plates for enumeration.

### Phenotypic observation of biofilms

#### Fluorescence microscopy assay

To microscopically visualize biofilm structural alterations, 13-mm-diameter glass coverslips were immersed in LB broth inoculated with 1 mL of 1×10^7^ CFU/mL *P*. *aeruginosa* PAO1 suspension in a flat-bottom 24-well polystyrene culture plate (Corning/Costar, USA), which was incubated statically at 37°C. For the inhibition assay described above, planktonic *P*. *aeruginosa* PAO1 suspensions were incubated with sub-MICs of baicalin (64, 128, and 256 μg/mL) simultaneously for various periods of time, as indicated. After treatment, non-adherent cells from each sample were removed by gently rinsing with sterile PBS, and the biofilms in each group were fixed with 4% paraformaldehyde solution (Sangon Biotech, Shanghai, China) for 30 min at room temperature. After rinsing with 2 mL of PBS, 5-(4,6-dichlorotriazinyl) aminofluorescein (5-DTAF, Thermo Fisher Scientific, Inc. Waltham, MA, USA) was added, and the samples were incubated at room temperature with shaking for 2 h according to the manufacturer’s instructions. Excess stain was then removed by washing with PBS. Fluorescent images were acquired using an upright microscope (BX53, Olympus, Japan) at 200× magnification and processed with cellSens Entry software (Olympus, Japan). The exposure times were 100–150 ms.

#### Confocal laser scanning microscopy (CLSM) protocol

To examine the biofilm-dispersing effects of baicalin and combinatorial treatment with antibiotics, 24-h and 96-h *P*. *aeruginosa* PAO1 biofilms were grown on glass coverslips as described in the above section. After 24 h of treatment with the tested agents, the remaining biofilms in each group were gently washed with sterile PBS, and then dead and live cells were detected using the fluorescent LIVE/DEAD BacLight™ bacterial viability kit (Molecular Probes, Invitrogen, USA) according to the manufacturer’s instructions. Viable *P*. *aeruginosa* PAO1 cells with intact membranes were stained fluorescent green, whereas those with damaged membranes were stained fluorescent red. Images of the stained biofilms were captured using a CLSM system (A1, Nikon, Japan) with a 488-nm argon laser and analyzed using Nikon NIS-Element software (Version 3.20, Nikon, Japan).

#### Scanning electron microscopy (SEM) protocol

Morphological changes in biofilms were also analyzed by SEM. Biofilm models were established and treated the same way as samples prepared for CLSM. After treatment and rinsing, the tested samples were fixed in 3% glutaraldehyde at 4°C overnight at room temperature. The coverslips were rinsed three times with sterile PBS for 10 min each and then dehydrated in an ethanol gradient series (30%, 50%, 70%, 80% and 90%) for 15 min per step. The samples were subsequently immersed in 100% ethanol (three times for ten min each) to prevent drying. Finally, all dehydrated samples were placed in a vacuum desiccator, coated with gold, and then observed using SEM (Tescan VEGA 3, Czech) at 30 kV.

### Detection of QS-controlled virulence factors

#### Supernatant preparation

QS-controlled virulence factors present in the supernatants of *P*. *aeruginosa* cultures, such as pyocyanin, rhamnolipid, LasA protease, LasB elastase and exotoxin A, were detected. Overnight cultures of *P*. *aeruginosa* PAO1 were adjusted to an OD_600_ of 2.0 in LB medium and then incubated with appropriate concentrations (64–256 μg/mL) of baicalin for 24 h. Cultures of *P*. *aeruginosa* PAO1 in the absence of agents, just containing 1% DMSO were served as a blank control. A *P*. *aeruginosa* PAO1 QS mutant deficient for *lasI-rhlI* was also cultured along with the treated and untreated *P*. *aeruginosa* PAO1 samples to serve as a negative control. After treatment, each group of culture samples was collected and centrifuged at 10,000 g for 15 min. The cell pellets were harvested by centrifugation and washed three times with sterile PBS for subsequent RNA extraction, and the supernatants were filtered via passage through a syringe filter (0.22 μm, Millipore) and subsequently stored at -80°C or used immediately to detect the remaining virulence factors. In addition, the prepared supernatants were used for detecting QS signaling molecules as described below under AHL determination.

#### LasA protease assay

LasA protease production in the supernatants of baicalin-treated or untreated samples was evaluated using an azocasein assay [32]. For each sample, 150 μL of filtered supernatant was mixed with 250 μL of 2% azocasein (Sigma-Aldrich, St. Louis, MO, USA) in 50 mM Tris-HCl buffer (pH = 7.8). The mixture was incubated at 4°C for 4 h. The reaction was terminated by the addition of 1.2 mL of 10% trichloroacetic acid followed by incubation at 4°C for 15 min and centrifugation at 10,000 rpm for 10 min. The supernatant was collected and mixed with 1.4 mL of 1 M NaOH. The relative protease activity was measured as the OD_440_ of the supernatant.

#### LasB elastase assay

The elastolytic activity of *P*. *aeruginosa* PAO1 propagated in different concentrations of baicalin was estimated by performing an elastin Congo red (ECR) assay using the procedure previously described by Ohman et al. [33]. Briefly, 1 mL of prepared supernatant filtrate was incubated with 1 mL of ECR (10 mg/mL in 100 mM Tris-HCl, pH = 7.5; 1 mM CaCl_2_) reaction buffer at 37°C for 16 h with shaking (200 rpm). The mixture was centrifuged at 3000 g for 10 min to remove insoluble ECR, and the OD_495_ was used to estimate elastase activity.

#### Pyocyanin assay

Pyocyanin pigment production was measured by performing a quantitative chemical assay as described by Essar et al. [34]. Briefly, 3 mL of culture supernatant prepared as described above was extracted with chloroform at a ratio of 3:2, followed by extraction with 1 mL of 0.2 M HCl. The absorbance of the upper red phase was measured using the OD_520_.

#### Rhamnolipid assay

An orcinol method was used to assess rhamnolipid levels in the cell-free supernatants of *P*. *aeruginosa* PAO1 cultures as previously described [35]. Briefly, a 1-mL supernatant sample (adjusted to pH 2 with HCl) was extracted twice with 1 mL of ethyl acetate. The organic phase was collected and evaporated overnight to dryness, and the residue was redissolved in 500 μL of water. Then, 900 μL of a solution containing 0.19% orcinol (in 53% [v/v] H_2_SO_4_) was added to 100 μL of each redissolved sample. After heating for 30 min in a water bath at 80°C, the samples were cooled to room temperature, and the absorbance was read at 421 nm. Rhamnolipid levels in the supernatant were calculated by comparing the data with the standard curve obtained for rhamnose standards between 0 and 300 μg/mL.

#### Detection of *P*. *aeruginosa* exotoxin A

Western blotting analysis [36] was performed to detect the production of *P*. *aeruginosa* exotoxin A (PEA) in culture supernatants. Briefly, the collected culture supernatant from each sample was dialyzed against 0.05 M Tris-HCl (pH = 8.0, Sigma) at 4°C and concentrated 10-fold in Minicon-B15 cells (Sigma-Aldrich, Merck Millipore, Darmstadt, Germany) with minimal (<5%) loss of exotoxin A. Before electrophoresis, protein-concentrated samples were heated in Protein Gel Loading Buffer (Sangon Biotech, Shanghai) to 100°C for 5 min, and 10 μL of preprocessed sample from each group was loaded and immediately electrophoresed in a Mini-Protean II vertical dual-cell apparatus (Bio-Rad) at room temperature at a constant voltage (110 V) for 1.5 h. The separated protein bands were visualized by staining with Coomassie brilliant blue R, and the gel containing PEA (indicated by the marker) was excised and dipped into Towbin system buffer (25 mM Tris-HCl pH 8.3, 192 mM glycine, 20% (v/v) methanol). Target proteins were transferred onto a PVDF membrane (Pall Corporation, USA) and blocked with 5% skim milk in TBS with 0.1% Tween 20 for 60 min at room temperature. The membrane was exposed to a goat polyclonal *Pseudomonas aeruginosa* exotoxin A primary antibody (1:500, Fitzgerald, MA, USA) and gently shaken for 1 h at room temperature. The membrane was then gently washed with sterile TBS three times for 10 min each. Next, the membrane was incubated with donkey anti-goat IgG H&L (conjugated to Alexa Fluor® 680) (1:10000, Abcam) secondary antibody for 30 min. The signal intensities of the bands were analyzed using the Odyssey infrared imaging system (Li-Cor) at 700 nm.

#### Motility assay

*P*. *aeruginosa* PAO1 swimming, swarming, and twitching motilities were measured according to Wu et al. [37]. To monitor swimming, diluted *P*. *aeruginosa* PAO1 at 1×10^7^ CFU/mL was point inoculated with a sterile toothpick onto plates containing 0.3% (w/v) Bacto agar, 0.2% casamino acids (w/v) and 30 mM glucose in the presence or absence of sub-MIC (256 μg/mL) baicalin. The plates were incubated for 24 h at 37°C. The migration distance around the incubation site was measured and compared to *P*. *aeruginosa* PAO1 and its deficient strain inoculated onto plates without baicalin. Swarming motility was measured on plates consisting of 0.4% (w/v) Bacto agar and LB supplemented with 0.5% (w/v) casamino acids, 0.5% (w/v) glucose and 256 μg/mL baicalin. Bacteria were positioned with sterile toothpicks into the centers of treated and untreated swarm plates and incubated at 37°C for 24 h. For the twitching assay, diluted *P*. *aeruginosa* PAO1 (1×10^7^ CFU/mL) was inoculated onto the bottom of a Petri dish containing various sub-MICs by stabbing a toothpick through a thin (2 mm) layer of LB medium supplemented with 0.2% casamino acids, 30 mM glucose, and 1.5% Bacto agar. After incubation for 24 h at 37°C, the agar was gently removed, and the Petri dish was air-dried. A 1% crystal violet solution was used to stain the plate agar interface for 10 min after removing the agar. The Petri dish was rinsed, and the crystal violet-stained twitching pattern was evaluated. The QS mutant *ΔlasI-ΔrhlI* was used as a negative control by applying the same procedure.

### Determination of AHLs

The two major QS molecules, 3-oxo-C12-HSL and C4-HSL, were extracted from 50-mL culture supernatants of *P*. *aeruginosa* PAO1 and its *lasR-rhlR*-deficient mutant, after the culture supernatants were incubated in the absence or presence of appropriate sub-MICs (64–256 μg/mL) of baicalin as described previously. Extraction was performed with ethyl acetate, which was acidified with 0.5% methanoic acid, and the resulting extracts were dried under nitrogen and quantified by high-performance liquid chromatography/electrospray mass spectrometry (HPLC/MS, Alliance 2695/Quattro Microsystem; Waters Corporation, Milford, MA, USA) according to methods adapted from Makemson et al. [38]. Mass spectra were observed for various m/z peaks of AHLs and for changes in their relative peak intensity (S1 Table). A standard curve generated from the pure compounds of 3-oxo-C12-HSL and C4-HSL was used to convert MS intensity data into corresponding concentrations in the culture supernatants.

### Analysis of QS gene expression

#### RNA extraction and cDNA preparation

To evaluate the effects of sub-MICs of baicalin, the expression levels of QS circuit genes, including *lasI*/*lasR* (*las* system), *rhlI*/*rhlR* (*rhl* system) and *pqsA*/*pqsR* (PQS system), were evaluated. Total RNA was extracted from *P*. *aeruginosa* PAO1 cultures that were incubated in the presence or absence of sub-MICs of baicalin for 24 h. *P*. *aeruginosa* PAO1 and its *lasI-rhlI*-deficient mutant were analyzed after growth under the same conditions. RNA extraction was performed using TRIzol reagent (Takara Holdings, Kyoto, Japan), and residual DNA was removed by treatment with DNase I (Thermo Fisher Scientific, Waltham, MA, USA) according to the manufacturer’s instructions. The purity and concentration of each RNA sample were determined spectrophotometrically at 260 and 260/280 nm, respectively, using a NanoDrop spectrophotometer (ND2000, Thermo Fisher Scientific, Waltham, MA, USA). RNA was then reverse-transcribed into complementary DNA (cDNA) using the RevertAid First Strand cDNA synthesis kit (Fermentas, Thermo Fisher Scientific, Waltham, MA, USA) following the manufacturer’s recommendations. The cDNA samples were used for subsequent real-time polymerase chain reaction (PCR) detection.

#### Quantitative real-time PCR

Quantitative real-time PCR was performed using a real-time PCR system (ABI 7500, Thermo Fisher Scientific, Waltham, MA, USA) with the specific primers listed in S3 Table. Amplification and expression were carried out in a total volume of 20 μL containing 12.5 μL of SYBR Premix Ex Taq with ROX Dye (Takara, China), 0.5 μL of each corresponding forward and reverse primer, 1 μL of cDNA, and 10.5 μL of ddH_2_O, as recommended by the manufacturer. The cycling parameters were as follows: holding stage of 95°C for 10 min; 40 cycles at 95°C for 15 s, 60°C for 60 s, and one melting curve stage of 95°C for 15 s, followed by 60°C for 60 s and 95°C for 15 s. All sample experiments were performed and analyzed in triplicate. The expression levels of the target genes were measured relative to the control sample and normalized to the expression of the endogenous reference gene (16S ribosomal RNA gene) between samples in parallel. The relative expression levels of target genes were calculated using the 2^-△△Ct^ method according to Schmittgen and Livak et al. [39].

### *C*. *elegans* survival assay

A *C*. *elegans*-*P*. *aeruginosa* infection model was used to evaluate the effects of baicalin on the pathogenicity of *P*. *aeruginosa*. Using a published protocol [1], gravid adult worms of the wild-type *C*. *elegans* N2 hermaphrodite strain were synchronized by hypochlorite treatment. The synchronized worms were then cultured to the L4 stage in an NGM plate at 20°C for the survival assay. A 7-cm brain heart infusion agar (BHI, Solarbio, Beijing, China) plate was prepared as *P*. *aeruginosa* infection agar. After pouring the plates, baicalin was added to the assay plates and mixed thoroughly to obtain a final concentration of 256 μg/mL before solidification. Next, 10 μL of an overnight culture of *P*. *aeruginosa* PAO1, *ΔlasI-ΔrhlI* mutant or *E*. *coli* OP50 was evenly spread onto BHI plates with or without baicalin supplementation and incubated at 37°C for 24 h to form a bacterial lawn. After equilibration to room temperature, 20 adult worms were picked and seeded onto plates, incubated at 20°C and observed every 24 h. The number of worms that survived was tabulated to generate a survival curve, the LT_50_ value (time required to kill 50% of the worms) was calculated, and the dead worms were removed from the assay daily.

### Effects of baicalin against *P*. *aeruginosa* infection in a mouse intraperitoneal foreign-body infection model

A mouse intraperitoneal foreign-body biofilm infection model was generated as previously described by Christensen et al. [20]. Healthy female BALB/c mice aged 8 weeks and weighing 18–22 g were purchased from the Laboratory Animal Center of Guangxi University (Nanning, People’s Republic of China) and used throughout this study. Before challenge, the mice were acclimated in specific pathogen-free (SPF) animal rooms for 5 days. This study protocol was conducted in accordance with the Guide for the Care and Use of Laboratory Animals published by the US National Institutes of Health (NIH publication 85–23, revised 1996) and was approved by the Animal Care and Use Committee of Guangxi Medical University, People’s Republic of China. Before operation, the mice were anesthetized with 50 mg/kg pentobarbital sodium to minimize the suffering and distress. The abdominal zone was shaved with a razor and disinfected with 75% ethanol. An incision of approximately 1 cm was made in the right groin area straight into the peritoneal cavity. Subsequently, a 4-mm silicone tube implant (with an outer diameter of 6 mm and an inner diameter of 4 mm; Lingyang, Zhejiang, China) was inserted into the peritoneal cavity with the use of sterile ophthalmic forceps. Before insertion, the implants were incubated in a *P*. *aeruginosa* PAO1 bacterial/normal saline solution at 37°C for 20 h with shaking at 110 rpm to allow for cell attachment and biofilm formation. The solution was prepared by dissolving a *P*. *aeruginosa* PAO1 bacterial pellet from a centrifuged overnight culture and resuspending it in 0.9% NaCl to achieve an OD_600_ of 0.1. The incision was then sutured with a 4–0 silk thread and healed without any complications. After challenge, the mice were divided into treatment groups (10 mice per group) and administered drugs. In the baicalin-treated group, the mice were given subcutaneous baicalin (100 mg/kg/t.i.d.), and in the antibiotic monotherapy groups, mice received an intraperitoneal injection of levofloxacin (25 mg/kg/b.i.d.), amikacin (20 mg/kg/b.i.d.), or ceftazidime (100 mg/kg/b.i.d.). In the combination treatment groups, the doses of baicalin and antibiotics were the same as those described for monotherapy. The daily therapeutic schedule was implemented for 3 consecutive days, and the mice were euthanized via the intraperitoneal injection of 20% pentobarbital at 3 mL/kg of body weight and cervical dislocation. The dosages of baicalin and antibiotics used in this study were based on previously published work [40] and our pilot experiments. Mice in the placebo group received an equivalent volume of vehicle. An uninfected silicone tube implant was also included as a negative control. Mice in another group were implanted with a silicon tube covered with *ΔlasI-ΔrhlI* mutant biofilm, which was prepared as described above. Mice were observed daily for functional behavior (i.e., fur quality, posture, state of activity and body weight) and abdominal symptoms (i.e., status of the abdominal incision). Mice were euthanized immediately after being found in a moribund state with a generalized infection as identified by the inability to remain upright, whether or not that was associated with labored breathing and cyanosis.

For bacteriological analysis, the silicone implants were removed from the peritoneal cavities of the mice and placed in Eppendorf tubes containing 2 mL of normal saline that was pre-cooled on ice. The tubes were then sonicated in an ultrasound bath for 10 min. Next, 100 μL of each sample solution was serially diluted 10-fold and spread onto an LB agar plate. The LB agar plates were incubated at 37°C for 24 h before determining the CFUs per implant.

The peritoneal lesion tissue surrounding the silicone implants was carefully separated, fixed in 10% formaldehyde solution (Sangon Biotech, Shanghai, China), and then embedded in paraffin wax and cut into slices, followed by hematoxylin and eosin staining.

Cytokine levels in the peritoneal cavity were estimated by performing an enzyme-linked immunosorbent assay (ELISA). The samples were prepared as described by Christensen et al. [20]. Briefly, the peritoneal cavities of the mice were flushed with 3 mL of normal saline with a syringe, and the collected fluid was stored at -80°C until use. Cytokine levels in each sample were quantified using commercial mouse interferon γ (IFN-γ) and interleukin 4 (IL-4) ELISA kits (Thermo Fisher Scientific, Waltham, MA, USA) following the manufacturer’s instructions. The absorbance was measured at 450 nm, and the concentrations were calculated from the linear portion of the standard curve.

### Statistical analysis

All experiments were repeated at least three times in duplicate to validate the reproducibility. All values are presented as the mean ± standard error. One-way analysis of variance was performed using SPSS 18.0 software (SPSS, Inc., Chicago, IL, USA) to compare differences between groups, followed by Dunn’s post hoc test. One-way repeated-measures ANOVA was used to analyze the differences in growth curves among the control group and the groups treated with different concentrations of baicalin. Survival was also examined using the Kaplan-Meier method, and differences were determined using the log-rank test. Significance was accepted when the *P*-value was less than 0.05. Graphs were constructed using GraphPad Prism software (Version 5.0; GraphPad Software, Inc., San Diego, CA, USA).

## Results

### MICs of the tested antibiotics and effects of baicalin on planktonic *P*. *aeruginosa* PAO1 growth *in vitro*

The MIC and MBC of baicalin against planktonic *P*. *aeruginosa* PAO1 are shown in Table 1; baicalin exerted no direct bactericidal effects and no completely bacteriostatic effects on planktonic *P*. *aeruginosa* PAO1 cells with both MBC and MIC >1024 μg/mL, at least at the concentration of 1024 μg/mL that we tested. *P*. *aeruginosa* PAO1 was sensitive to three of the tested antibiotics. Antibiotic MICs against the quality control strain *P*. *aeruginosa* ATCC27853 were also obtained in parallel (shown in S2 Table), and all results were within the range recommended by the CLSI.

**Table 1:** Susceptibility of *P*. *aeruginosa* PAO1 to antimicrobial agents.

Antimicrobial agents	MIC	MBC
Baicalin (μg/mL)	>1024	>1024
Levofloxacin (μg/mL)	0.5	1
Amikacin (μg/mL)	1	8
Ceftazidime (μg/mL)	1	2

**Fig 1:**
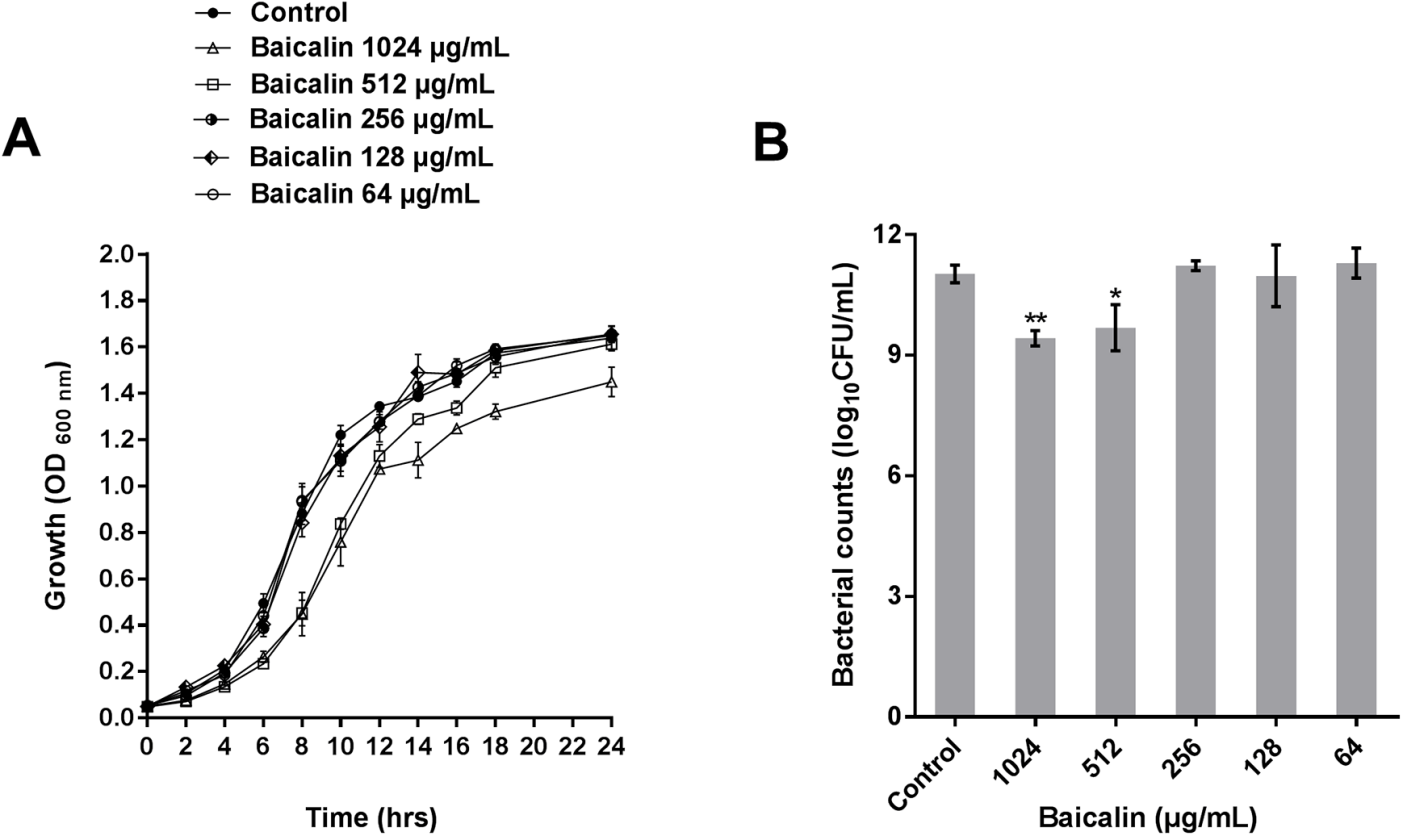
Effects of baicalin on the growth of *P*. *aeruginosa* PAO1. Growth curves (**A**) and bacterial colony counts (**B**) of *P*. *aeruginosa* PAO1 treated with varying concentrations of baicalin (64–1024 μg/mL) in Luria-Bertani broth at 37°C for 24 h. Experiments were performed in triplicate; the error bars represent the standard error of the OD_**600**_ value for each time point in the growth curves and the standard error for the number of CFUs after 24 h treatment. * and ** indicate *P*-values <0.05 and <0.01, respectively, with respect to the control (no baicalin).

*P*. *aeruginosa* PAO1 viability in the presence of sub-MICs of baicalin was assessed to determine whether any effects of baicalin were caused by the modification of cell function as opposed to bactericidal or bacteriostatic effects. As indicated by the growth curve in Fig 1A, baicalin at concentrations ≤256 μg/mL did not significantly inhibit *P*. *aeruginosa* PAO1 kinetic planktonic cell growth when compared with the control group (*P >*0.05). However, at concentrations ≥512 μg/mL, the cell density and kinetic growth of planktonic *P*. *aeruginosa* PAO1 were significantly inhibited (*P*<0.05). The inhibition of viability was further quantified by plating the cultures and counting CFUs after baicalin treatment for 24 h. Only cultures containing ≥512 μg/mL baicalin demonstrated significantly fewer CFUs at 24 h than the control group (*P*<0.05). The above result indicated that concentrations ≥512 μg/mL may have affected other bacterial processes, such as bacterial growth, in addition to QS. Therefore, sub-MICs ≤256 μg/mL were selected as the concentrations for testing in the subsequent anti-biofilm and anti-QS experiments.

### Baicalin prevents *P*. *aeruginosa* biofilm formation

**Fig 2:**
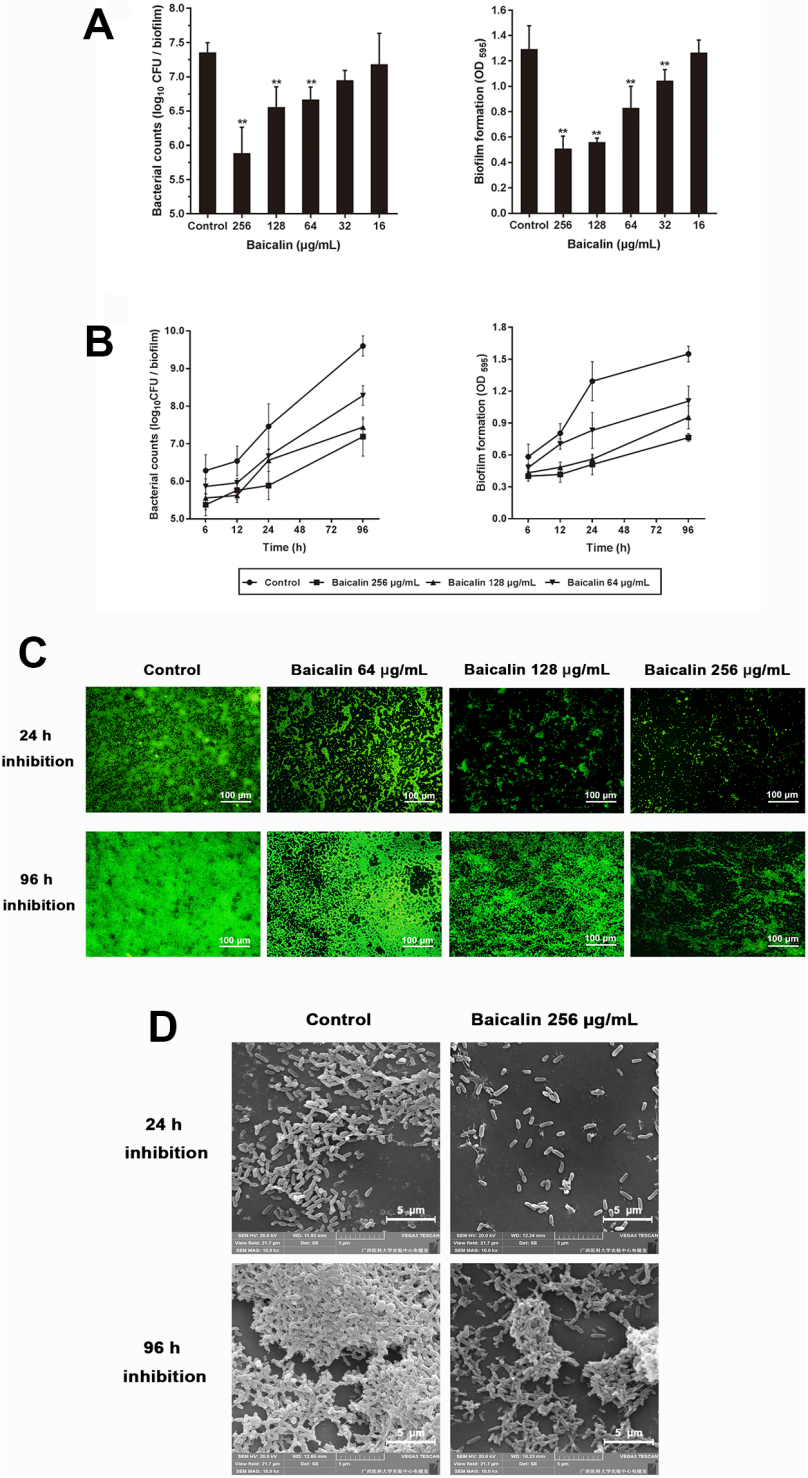
Inhibitory effects of baicalin on *P*. *aeruginosa* PAO1 biofilm formation. In the dose-dependent analysis (**A**), bacterial suspensions were seeded in 96-well flat-bottomed polystyrene microtiter plates exposed to sub-MICs of baicalin (16, 32, 64, 128, and 256 μg/mL) for 24 h, and biofilm mass formation and bacterial counts were quantified in triplicate. Values represent the mean ± standard deviation. *P<0.05 and ^**^P<0.01 compared with the control group. In the time-dependent analysis (**B**), biofilm mass formation and bacterial counts were monitored after exposing biofilms to sub-MICs (64, 128, and 256 μg/mL) of baicalin for 6, 12, 24 or 96 h. Biofilm architecture after 24 h and 96 h of baicalin inhibition was examined by fluorescence microscopy (200× magnification) (**C**) and SEM (10,000× magnification) (**D**).

To evaluate the potential anti-biofilm activity of baicalin, we first tested whether baicalin at sub-MICs (16, 32, 64, 128, 256 μg/mL) inhibited *P*. *aeruginosa* PAO1 biofilms after 24 h of treatment. Sub-MICs (64, 128, 256 μg/mL) of baicalin significantly reduced cell attachment and biofilm biomass development in a dose-dependent manner (Fig 2A). Baicalin at 32 μg/mL exerted activity against *P*. *aeruginosa* PAO1 biomass formation, but concentrations below 64 μg/mL had no obvious effects on biofilm bacterial counts. *P*. *aeruginosa* biofilm formation includes the following stages: initial planktonic cell attachment, cell proliferation, accumulation in multilayer cell clusters, and final formation of the bacterial community in a complex polymeric matrix. We further investigated whether sub-MICs of baicalin exert inhibitory effects against *P*. *aeruginosa* biofilms at different time points (6, 12, 24 or 96 h). Baicalin had time-dependent effects on the inhibition of viable cell accumulation and biofilm mass formation (Fig 2B). These effects became more pronounced after prolonged treatment (24 h or 96 h). Similar findings were observed upon biofilm visualization using fluorescence microscopy (Fig 2C): baicalin inhibited *P*. *aeruginosa* biofilm formation in a concentration-dependent manner, with fewer bacteria adhering to the coverslip surface and decreased polysaccharide production compared to the control, regardless of whether the cultures were grown for 24 or 96 h. Examination of the architecture of biofilms that were pre-exposed to baicalin indicated a dramatic reduction in the amount of biofilm attached to the coverslip surface after treating 24-h or 96-h *P*. *aeruginosa* biofilms with baicalin (256 μg/mL), with fewer multilayer cell clusters (Fig 2D).

### Baicalin in combination with certain antibiotics effectively disrupts established *P*. *aeruginosa* biofilms

**Fig 3:**
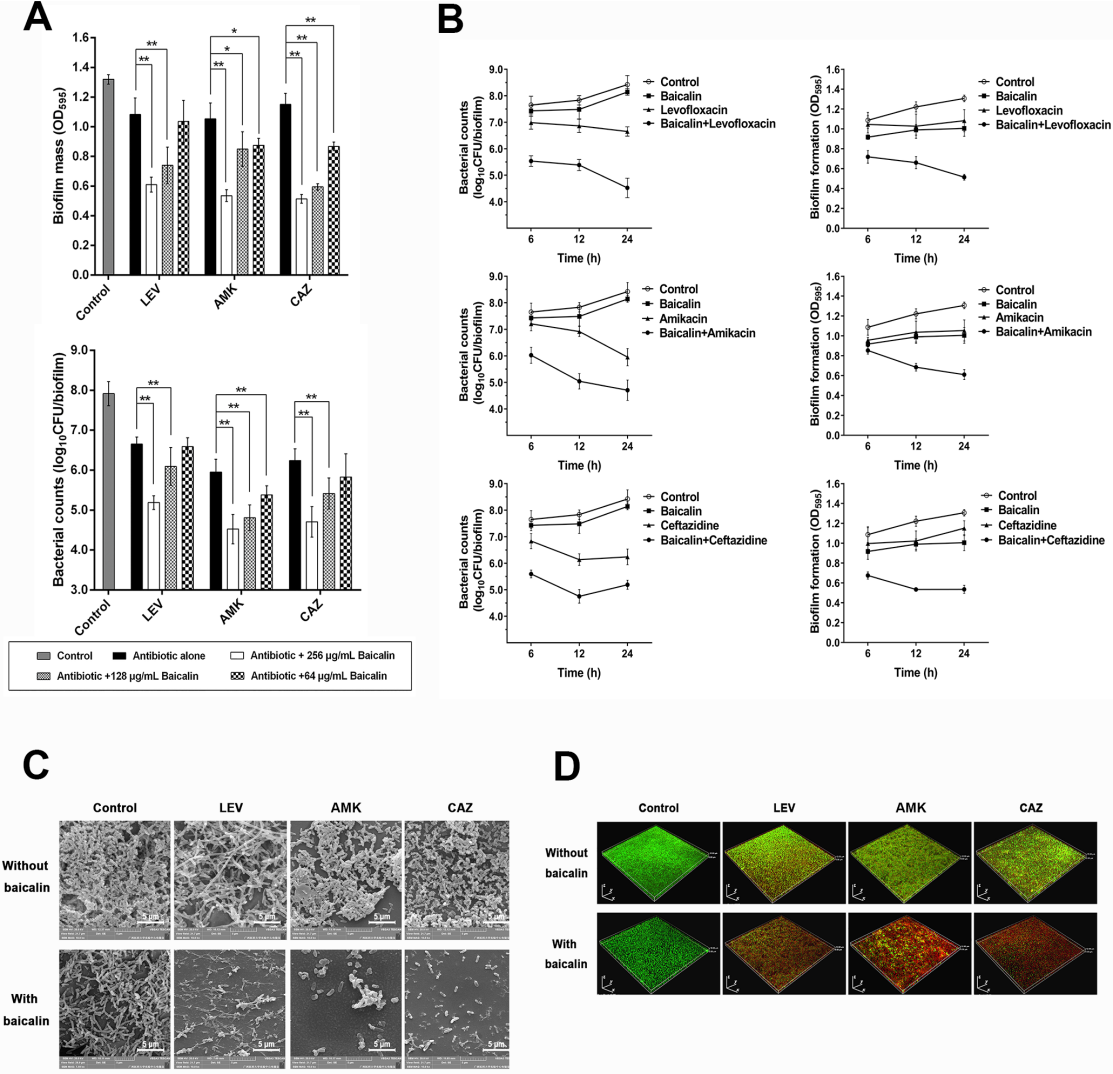
Baicalin as a co-treatment in combination with three conventional antibiotics to treat 24-h *P*. *aeruginosa* PAO1 biofilms. (A) *P*. *aeruginosa* PAO1 biofilm mass and bacterial counts were quantified after treating pre-existing 24-h biofilms with levofloxacin (1 μg/mL), tobramycin (8 μg/mL) and ceftazidime (2 μg/mL) alone or in combination with various sub-MICs (64, 128, and 256 μg/mL) of baicalin for 24 h. (B) Biofilms were exposed to baicalin (256 μg/mL), levofloxacin (1 μg/mL), tobramycin (8 μg/mL), ceftazidime (2 μg/mL) or a baicalin/antibiotic mixture for 6, 12 and 24 h, and changes in biofilm mass formation and bacterial counts were monitored over time. Experiments were performed in triplicate, and values represent the mean ± standard deviation. *P<0.05 and ^**^P<0.01 compared with the vehicle control group. (C) Images show 24-h biofilms observed by SEM (10,000× magnification). (D) Three-dimensional reconstructions of 24-h *P*. *aeruginosa* PAO1 biofilms after staining with the LIVE/DEAD viability kit were created using CLSM (200× magnification).

**Fig 4:**
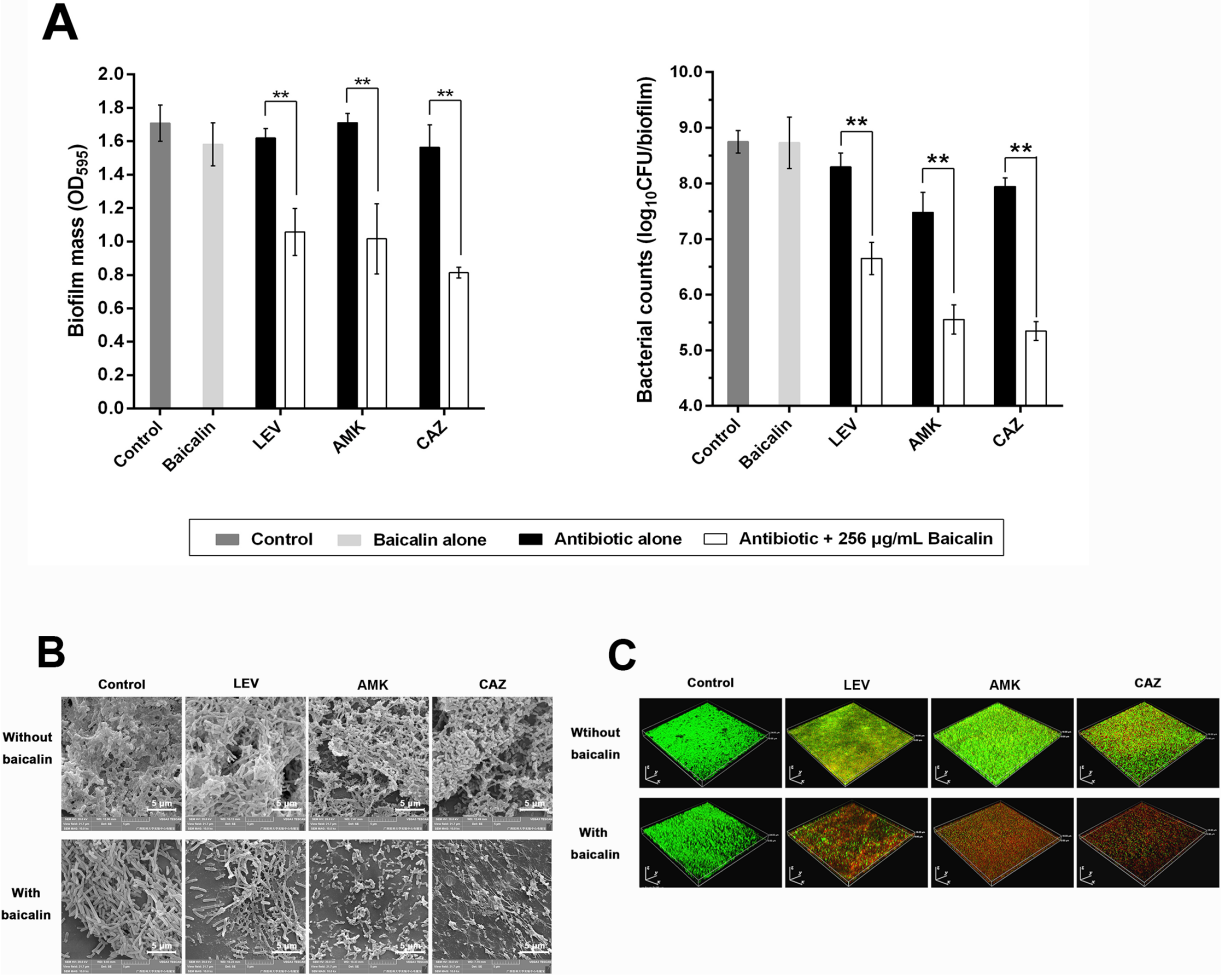
Baicalin as a co-treatment in combination with three conventional antibiotics to treat 96-h *P*. *aeruginosa* PAO1 biofilms. (A) *P*. *aeruginosa* PAO1 biofilm mass and bacterial counts were quantified after treating pre-existing 96-h biofilms with sub-MIC (256 μg/mL) of baicalin for another 24 h. Experiments were performed in triplicate, and values represent the mean ± standard deviation. ^**^P<0.01 compared with the vehicle control group. (B) Images show 96-h biofilms observed by SEM (10000× magnification). (C) Three-dimensional reconstructions of 96-h *P*. *aeruginosa* PAO1 biofilms were created using CLSM (200× magnification).

Alone, baicalin at sub-MICs (64, 128, and 256 μg/mL) had little effect on the existing biomass of 24-h *P*. *aeruginosa* PAO1 biofilms after treatment for 24 h. Baicalin at 256 μg/mL exhibited a minor but significant direct biofilm dispersion effect, while bacterial counts did not appear to be affected (S1 Fig), largely because baicalin at sub-MIC levels did not exert direct bactericidal effects on pre-existing *P*. *aeruginosa* in the biofilm. Further, treatment for 24 h with three different types of antibiotics, specifically levofloxacin, amikacin, or ceftazidime, at MBCs did not result in notable decreases in either biofilm mass or numbers of viable cells. Interestingly, the combination of 256 μg/mL baicalin and the three antibiotics at their MBCs resulted in enhanced *P*. *aeruginosa* PAO1 biofilm loss, in the form of marked reductions in both biofilm biomass and bacterial counts, compared with the corresponding single antibiotic treatment groups. A concentration-dependent analysis indicated that 128 μg/mL baicalin in combination with antibiotics also exerted synergistic effects on *P*. *aeruginosa* biofilm eradication, whereas 64 μg/mL baicalin in combination with levofloxacin did not impact the colony counts or biomass of *P*. *aeruginosa* PAO1 biofilms, nor did that treatment in combination with ceftazidime. Thus, antibiotics at their MBCs combined with baicalin at a higher sub-MIC were more efficient at disrupting *P*. *aeruginosa* PAO1 biofilms than when applied individually (Fig 3A). Additionally, sufficient amounts of sub-MIC baicalin were required to retain anti-biofilm efficacy in combination with antibiotics. Next, we tested whether the combination of baicalin/antibiotics disrupted preformed biofilms in a time-dependent manner. These combinations exerted more pronounced effects in terms of decreased biofilm mass and viable cell counts (Fig 3B) compared with baicalin or antibiotics alone after short- or long-term treatment. The treatment of 96-h *P*. *aeruginosa* PAO1 biofilms, which are considered more mature and more resistant to antibiotics than 24-h biofilms, also resulted in significant reductions in biofilm mass and viable cell counts after combined baicalin (256 μg/mL)/antibiotic treatment compared with individual reagents alone (Fig 4A). Visualization of 24-h and 96-h *P*. *aeruginosa* biofilms by SEM revealed reduced extracellular matrix following baicalin treatment. Baicalin or antibiotics alone did not eradicate *P*. *aeruginosa* from pre-existing biofilms. However, combined treatment with baicalin and antibiotics was more effective at disrupting biofilms because very few *P*. *aeruginosa* PAO1 cells remained, and those that were present showed a scattered distribution (Figs 3C and 4B). Similar to the results of the SEM analyses, 24-h and 96-h *P*. *aeruginosa* biofilms exposed to baicalin, antibiotics or their combination were stained and processed using CLSM. Representative CLSM images are shown in Fig 3D and Fig 4C. The antibiotics barely penetrated the biofilms to kill bacteria, as evidenced by the dominant green fluorescence in individual antibiotic groups that was equivalent to the control group. The sub-MIC of baicalin also exerted no bactericidal effects on *P*. *aeruginosa* and primarily exhibited green fluorescence, while red fluorescence (representing dead cells) prevailed in the baicalin/antibiotic combination groups.

### Effects of baicalin on QS-controlled *Pseudomonas aeruginosa* virulence

**Fig 5:**
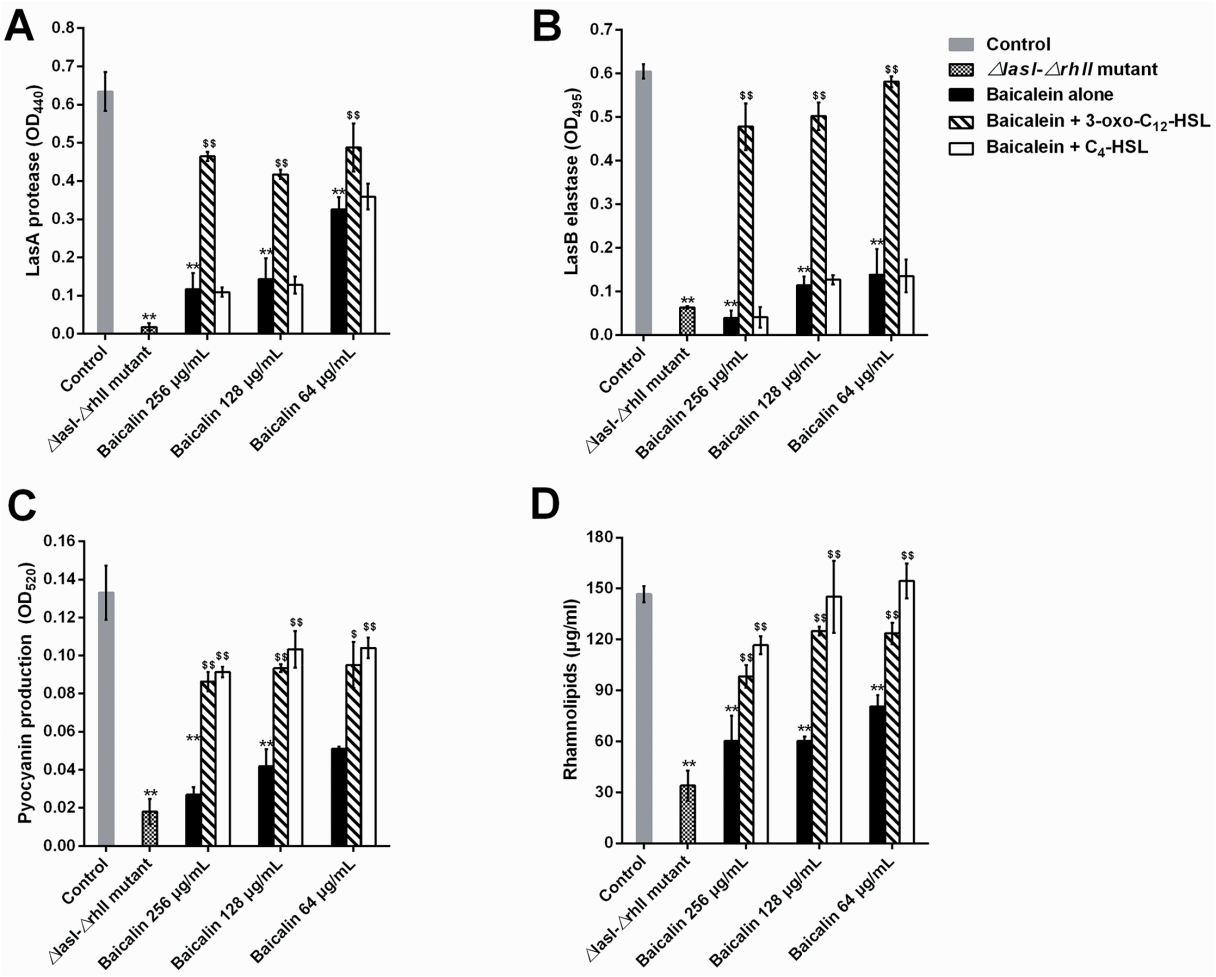
Inhibitory effects of sub-MICs of baicalin on the production of QS-regulated extracellular virulence factors in *Pseudomonas aeruginosa* PAO1 culture supernatants. Virulence factor activity, including LasA protease (A), LasB elastase (B), pyocyanin (C), and rhamnolipid activities (D), was analyzed. Error bars indicate the standard deviations of three measurements. ^**^P<0.01 compared with the untreated control group. ^$ $^*P*<0.01 compared with the same concentration of baicalin alone.

Four QS-related virulence factors, LasA protease, LasB elastase, pyocyanin and rhamnolipid, were first analyzed spectrophotometrically to assess the effects of baicalin on the QS system of *P*. *aeruginosa*. The production of these four QS-relevant virulence factors was significantly reduced by sub-MICs (64, 128, and 256 μg/mL) of baicalin in a dose-dependent manner: 48.6–81.6% suppression for LasA protease, 77.1–93.6% suppression for LasA protease, 61.7–79.7% suppression for pyocyanin, and 45.1–58.9% suppression for rhamnolipid. In particular, sub-MICs of baicalin reduced LasB elastase activity in *P*. *aeruginosa* PAO1 almost to the levels found in the *ΔlasI-ΔrhlI* double mutant. To further investigate whether the inhibitory effects of baicalin were reversed by supplementation with exogenous AHLs, cultures were treated with synthetic 3-oxo-C12-HSL (0.13 μM) or C4-HSL (5.2 μM; Sigma-Aldrich) during early logarithmic growth. These doses were maximal autoinducer concentrations for *P*. *aeruginosa* PAO1 [41]. Interestingly, the addition of exogenous 3-oxo-C12-HSL relieved the inhibitory effects of baicalin on the secretion of all four detected extracellular virulence factors described previously, whereas C4-HSL significantly antagonized the inhibitory effects of baicalin in terms of pyocyanin and rhamnolipid production (Fig 5).

**Fig 6:**
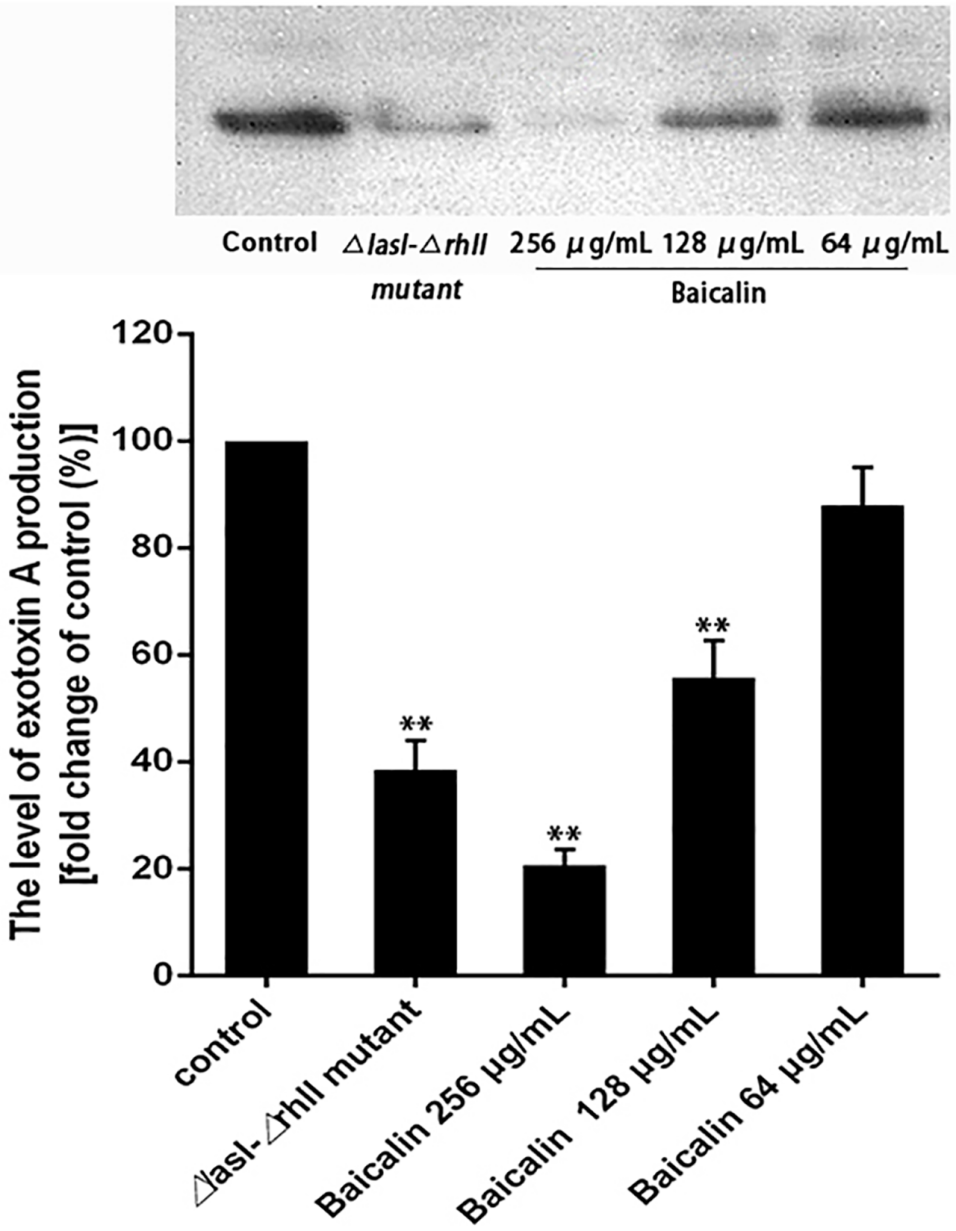
Effects of baicalin on PEA production detected by western blotting. Top image: Western blot analysis of PEA in the control group and in groups treated with sub-MICs of baicalin (64, 128, and 256 μg/mL) for 24 h. Bottom image: band intensity quantitation based on densitometry. ^**^P<0.01 versus the control group. Data are shown as the average of three experiments.

Western blot analysis was performed after baicalin treatment to determine whether sub-MIC levels of baicalin affected *P*. *aeruginosa* exotoxin A (PEA) production levels. As shown in Fig 6, the extent of PEA production decreased with increasing concentrations of baicalin (64, 128, and 256 μg/mL). In particular, treatment with 256 μg/mL baicalin produced a marked reduction to approximately 20.7% of that in the control group. Additionally, this severe reduction was much lower than in the *ΔlasI-ΔrhlI* double mutant. However, there was no significant reduction in response to 64 μg/mL baicalin treatment.

**Fig 7:**
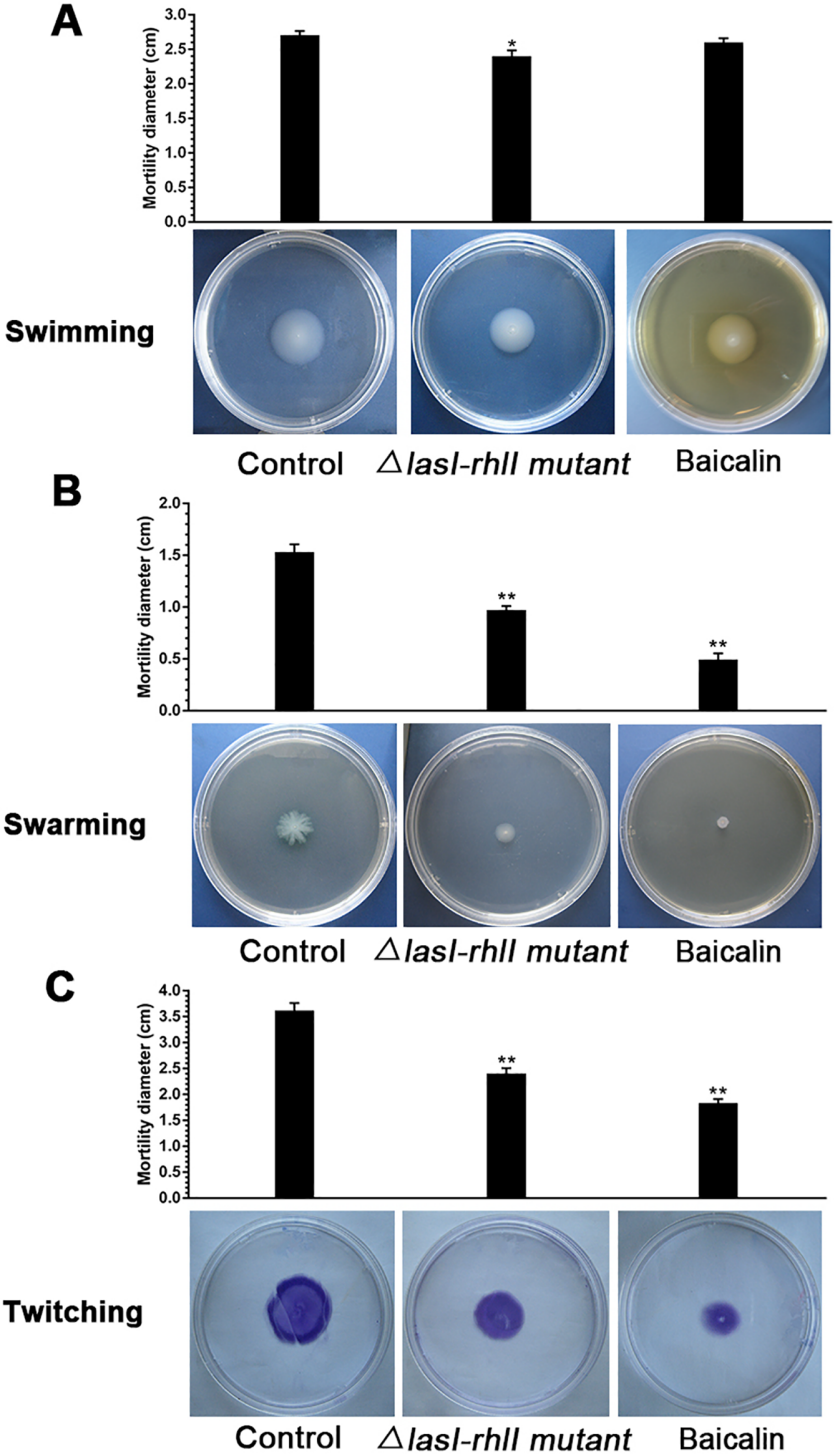
Effects of baicalin on *P*. *aeruginosa* PAO1 motility. Three motility assays were conducted on Plates Containing Different Concentrations of Agar in the Absence of Baicalin (Untreated Control) or Containing 256 μg/mL Baicalin. (A) Swimming, (B) swarming, and (C) twitching motility diameters were measured using a caliper. The data represent the average values from three independent experiments performed in duplicate. Values are the mean ± standard deviation. *P<0.05 and **P<0.01 compared with the untreated control group.

The effects of baicalin on the swimming, swarming and twitching motilities of *P*. *aeruginosa* were determined by inoculating overnight cultures of *P*. *aeruginosa* PAO1 onto motility plates. In the absence of baicalin supplementation, the swarming and twitching motility zones for *P*. *aeruginosa* PAO1 were 1.53±0.22 cm and 3.62±0.43 cm, respectively. The presence of 256 μg/mL baicalin resulted in significant decreases (*P*<0.05) in both swarming and twitching motility to 0.49±0.18 cm and 1.83±0.23 cm, respectively. These reductions were lower than the motility observed for the *ΔlasI-ΔrhlI* mutant in its normal state (0.97±0.12 cm for swarming and 2.41±0.31 cm for twitching). However, baicalin did not inhibit the swimming ability of *P*. *aeruginosa* PAO1 compared with the control (2.71±0.19 cm); at best, it exerted a weak inhibitory effect (2.60±0.18 cm) that was not statistically significant (*P*>0.05) (Fig 7).

### Effects of baicalin on the production of QS signaling molecules

**Fig 8:**
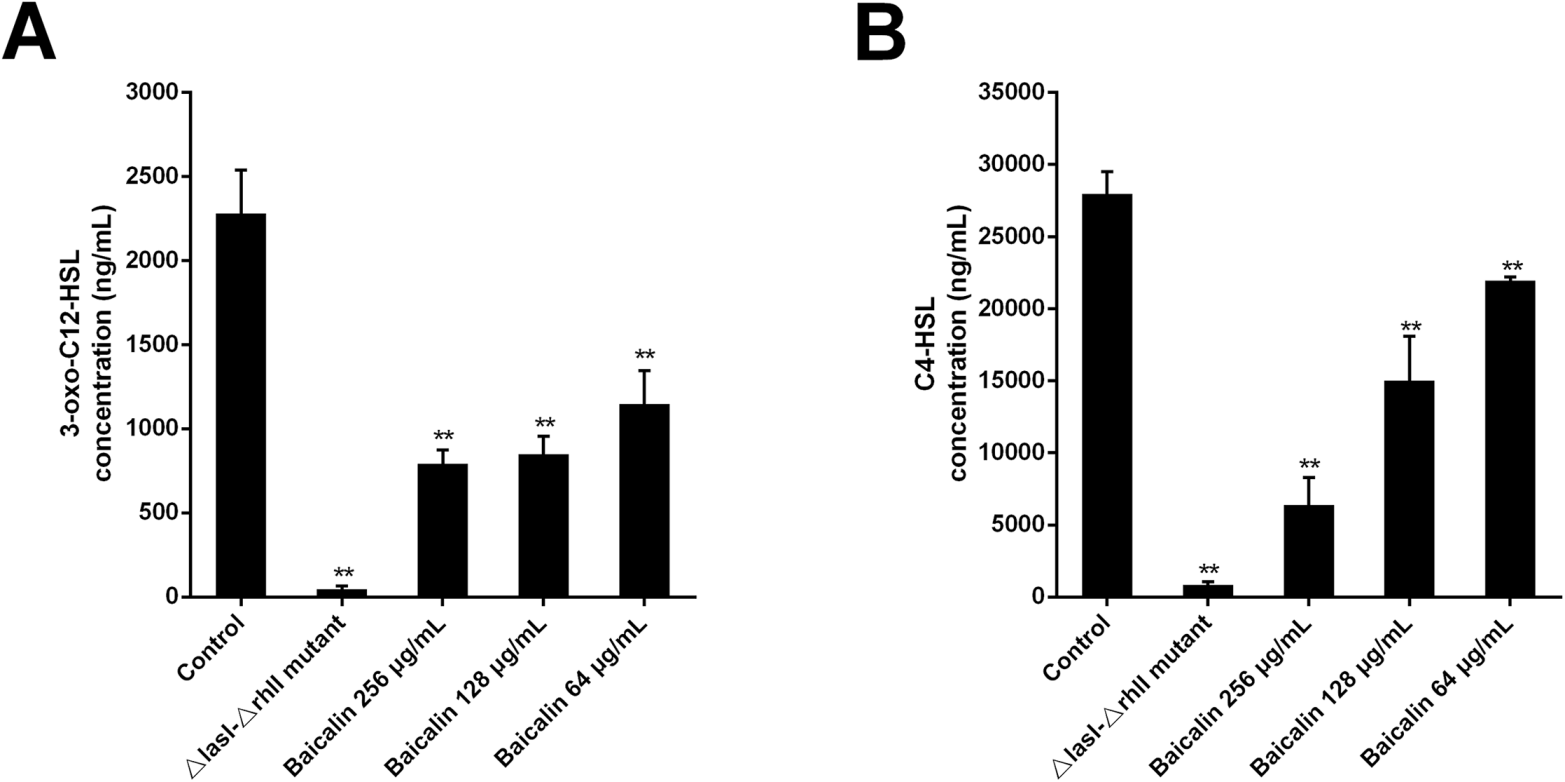
**Effects of Sub-MICs of Baicalin on the Production of QS Signaling Molecules [3-oxo-C12-HSL (A) and C4-HSL (B)] Extracted from *P*. *aeruginosa* PAO1 Culture Supernatants and Analyzed by HPLC/MS.** The experiment shown is representative of three independent tests. **P<0.01 versus untreated control. Error bars indicate the standard deviation of three parallel measurements.

AHL molecule production was assessed by performing an HPLC/MS assay. Positive controls consisting of untreated *P*. *aeruginosa* PAO1 cultures yielded the highest levels of 3-oxo-C12-HSL (2281.2±256.3 ng/mL) and C4-HSL (27961.3±892.9 ng/mL) in the culture supernatants. In contrast, the *ΔlasI-ΔrhlI* mutant strain barely produced measurable signaling molecules. When *P*. *aeruginosa* PAO1 cultures were grown in the presence of sub-MICs of baicalin (64, 128, and 256 μg/mL), 3-oxo-C12-HSL levels were reduced by 49.6%, 62.8% and 65.3%, respectively, which were all significant relative to the control (*P*<0.01, Fig 1A). Significant decreases in the C4-HSL levels, 77.2%, 46.3% and 21.6% (*P*<0.01) in the presence of 64, 128, and 256 μg/mL baicalin, respectively, were also observed (Fig 8).

### Expression of QS-regulated genes

**Fig 9:**
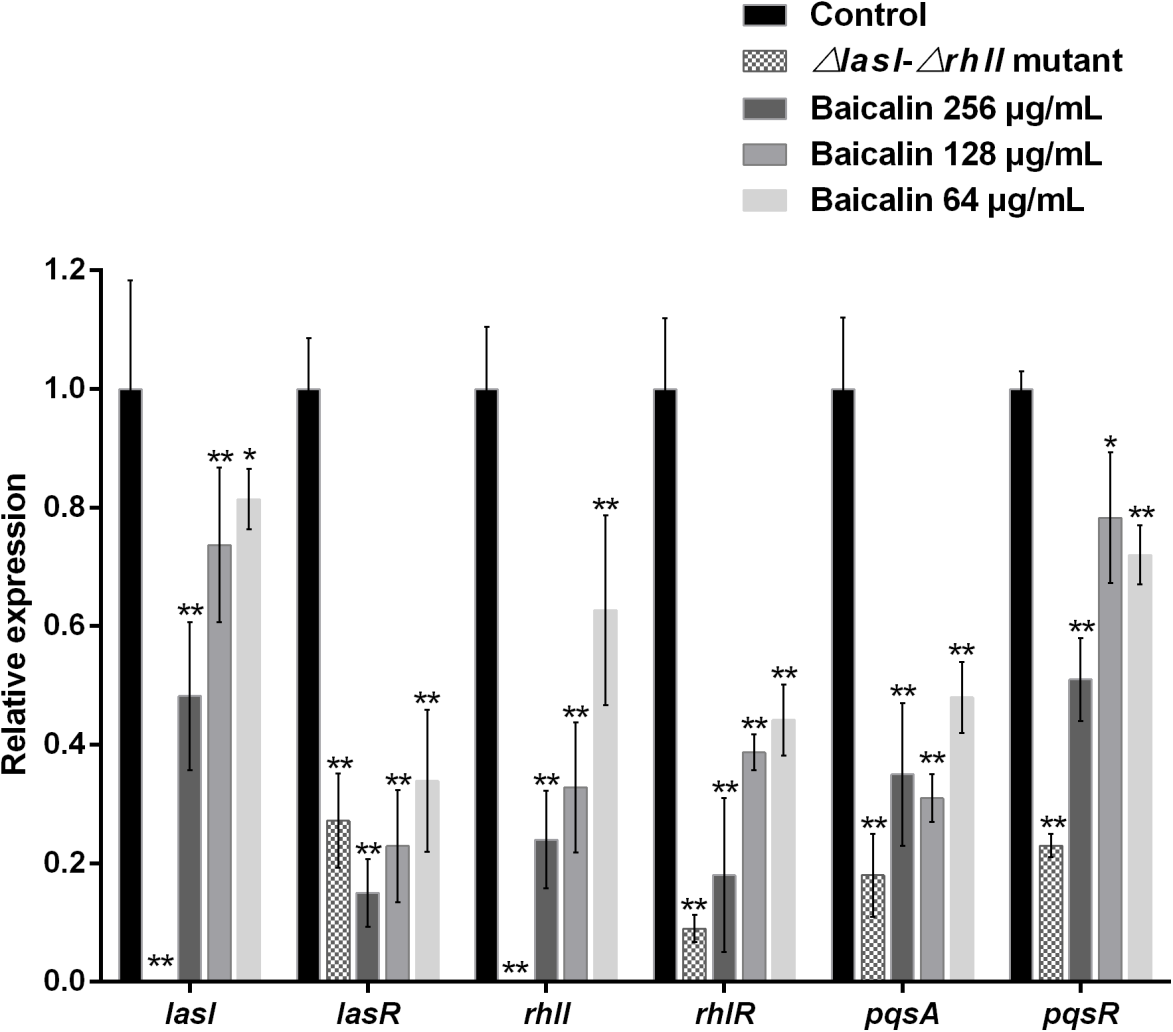
Relative expression levels of QS-regulated genes in the presence of sub-MICs of baicalin (64, 128, and 256 μg/mL) as determined by real-time quantitative PCR. *P<0.05 and **P<0.01 versus the untreated control. Error bars indicate the standard deviation of three parallel measurements.

The expression of key QS-regulated genes (*lasI*, *lasR*, *rhlI*, *rhlR*, *pqsA* and *pqsR*) was assessed in the presence of sub-MICs of baicalin-treated and untreated *P*. *aeruginosa* PAO1 using real-time quantitative PCR. Gene expression was also assessed for the *ΔlasI-ΔrhlI* mutant strain as a negative control. The standard curve for the housekeeping gene 16S rRNA indicated that all tested samples fell on the same line with an *R*^2^ value of 0.997. Furthermore, all standard curves for the four target genes had R^2^ values ranging from 0.978 to 0.994. Melting curves obtained with *16S rRNA*, *lasI*, *lasR*, *rhlI*, *rhlR*, *pqsA* and *pqsR* indicated that *P*. *aeruginosa* standards and samples had the same melting profiles, which were characterized by the formation of pure gene amplicons and no primer dimers. According to the real-time PCR data, baicalin at 256 μg/mL significantly repressed *lasI*, *lasR*, *rhlI*, *rhlR*, *pqsA* and *pqsR* transcription levels by 51.8%, 85.2%, 76.0%, 82.2%, 65.7%, and 49.5%, respectively, compared with the untreated control (*P*<0.01). Moreover, these inhibitory effects against the four target genes (*lasI*, *lasR*, *rhlI*, *rhlR*) involved in the *las* or *rhl* system were enhanced in response to increasing concentrations of baicalin (64, 128, and 256 μg/mL), while 128 μg/mL baicalin resulted in the most significant reduction in *pqsA* gene expression. Taken together, sub-MICs of baicalin may globally genetically inhibit not only the *las* and *rhl* systems but also the PQS system in *P*. *aeruginosa* (Fig 9).

### Exogenous supplementation with baicalin delays *C*. *elegans* death due to *P*. *aeruginosa* PAO1

**Fig 10:**
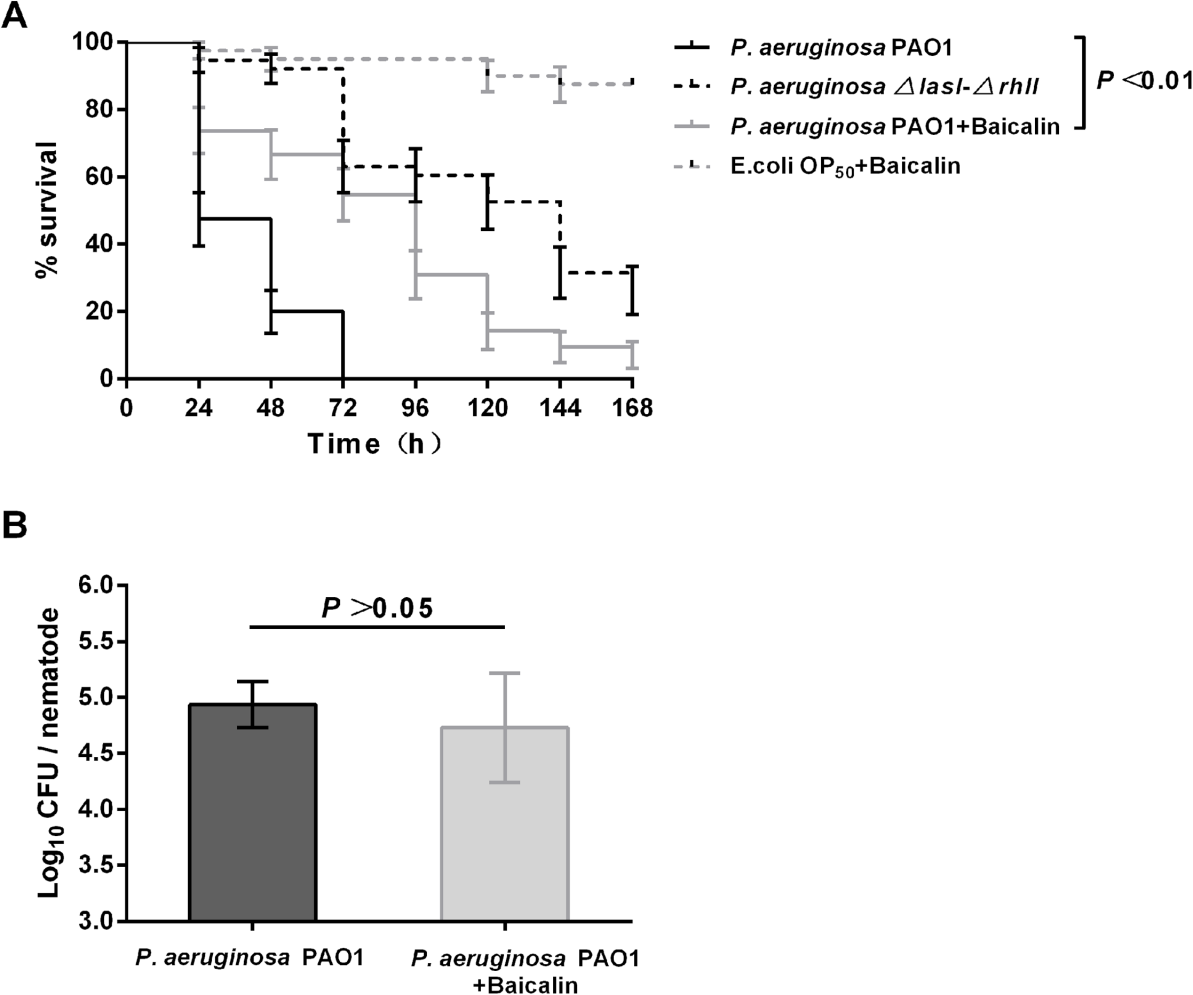
Effects of baicalin on the survival of *C*. *elegans* infected with *P*. *aeruginosa* PAO1. (**A**). Kaplan-Meier survival curve of *P*. *aeruginosa-*infected *C*. *elegans*. (**B**). Colony counts of *P*. *aeruginosa* PAO1 recovered from worms fed untreated and 256 μg/mL baicalin-treated *P*. *aeruginosa*. After 24 h, the nematodes were washed to remove bacteria from the integument and ruptured to recover bacteria from the digestive tracts. Bacterial loads were determined by plating on the appropriate selective medium and counting colonies. Data were obtained from three experiments.

We further investigated the anti-virulence and preventive effects of baicalin at sub-MICs on the ability of *P*. *aeruginosa* PAO1 to kill *C*. *elegans*. After exposure to *P*. *aeruginosa* PAO1 under standard conditions of the slow-killing assay, the infected *C*. *elegans* showed a nearly 52% decrease in mortality over 24 h of incubation (LT_50_ is 24 h) that subsequently led to complete death (100%) within 72 h. Comparing the Kaplan-Meier survival curves, treatment of infected *C*. *elegans* with sub-MIC baicalin (256 μg/mL) significantly improved survival (*P*<0.01) and increased the LT_50_ to 96 h. Worms infected with *P*. *aeruginosa ΔlasI-ΔrhlI*, which is deficient in QS and considered hypovirulent, had an LT_50_ of 144 h, which was markedly longer than that obtained for wild-type *P*. *aeruginosa* (Fig 10A). In addition, the living state of *C*. *elegans* fed *E*. *coli* OP50 in the presence of baicalin at a sub-MIC of 256 μg/mL was similar to that of *C*. *elegans* fed *E*. *coli* OP50 without baicalin, showing proper egg laying, a regular life cycle (2.5 days at 25°C), normal motility and improved survival (mortality less than 5%) throughout the assay, consistent with an absence of baicalin toxicity. The bacterial load inside the nematode was determined according to the protocol described by Garsin et al. [42]. Bacterial loads in the worms after 24 h of feeding on *P*. *aeruginosa* PAO1 were not significantly different (*P* = 0.233, 4.94±0.21 log_10_ CFU/worm in untreated worms and 4.73±0.49 log_10_ CFU/worm in treated worms) (Fig 10B), indicating an attenuation of the virulence of *P*. *aeruginosa* PAO1 colonizing the worm gut with no effect on viability.

### Effects of baicalin on the clearance of P. aeruginosa in a mouse foreign-body infection model

Based on previous *in vitro* findings demonstrating the effects of baicalin on *P*. *aeruginosa* biofilm formation and the QS system, the effects of baicalin were further investigated in a *P*. *aeruginosa*-related foreign-body infection model *in vivo*. In our pilot study, we assessed the pharmacokinetic characteristics of baicalin in non-infective BALB/c mice. For single subcutaneous bolus doses of 25, 50, and 100 mg/kg baicalin, the maximum plasma concentrations of baicalin (*C*_*max*_) were 59.71, 91.61 and 212.64 μg/mL, respectively. The values of *AUC* (0-∞) were 196.99, 267.35 and 666.18 μg/mL* h, respectively. The mean residence times [MRT (0-∞)] were 3.01, 3.12 and 3.00 h, respectively (S2 Fig). Thus, 100 mg/kg was selected as the dose for baicalin treatment, as it was within the effective range in which baicalin was effective against biofilm formation and QS by *P*. *aeruginosa*. In the present study, we first evaluated the biofilm bacterial burden in the inserted silicone implants after a 3-day treatment course with baicalin, antibiotics or their combination. *P*. *aeruginosa* PAO1 treated with a combination of baicalin and antibiotics showed significant differences in recovered CFU counts compared with the untreated control group. Significant differences in clearance were also observed between the baicalin/antibiotic combination treatment groups and the corresponding antibiotic single-treatment groups. Furthermore, we identified a significant difference in clearance between the baicalin single-treatment group and the control group (Table 2). Consistent with previous reports from other groups, the QS-deficient *P*. *aeruginosa* strain was more rapidly cleared from the implants by the immune system (CFU counts for *P*. *aeruginosa ΔlasI-ΔrhlI* in the infected group were 4.39±0.65, *P*<0.01 compared with the untreated control group).

**Table 2:** Clearance of implants pre-colonized with wild-type *P*. *aeruginosa* PAO1 inserted in the peritoneal cavities of BALB/c mice treated with various agents alone or in combination for 3 days. Results are expressed as the mean ± standard deviation. *P<0.01 compared with the vehicle control group without baicalin treatment. ^&^*P*<0.05 and ^▲^*P*<0.01 when the combination treatment group was compared with the antibiotic treatment alone.

Group	Bacterial counts (Log_10_ CFU/implant)	
	— Baicalin	+ Baicalin
Control	6.24±0.66	5.38±0.50*
LEV	5.22±0.42*	4.46±0.61*^▲^
AMK	4.83±0.30*	4.10±0.87*^&^
CAZ	5.06±0.76*	3.89±0.62*^▲^

**Fig 11:**
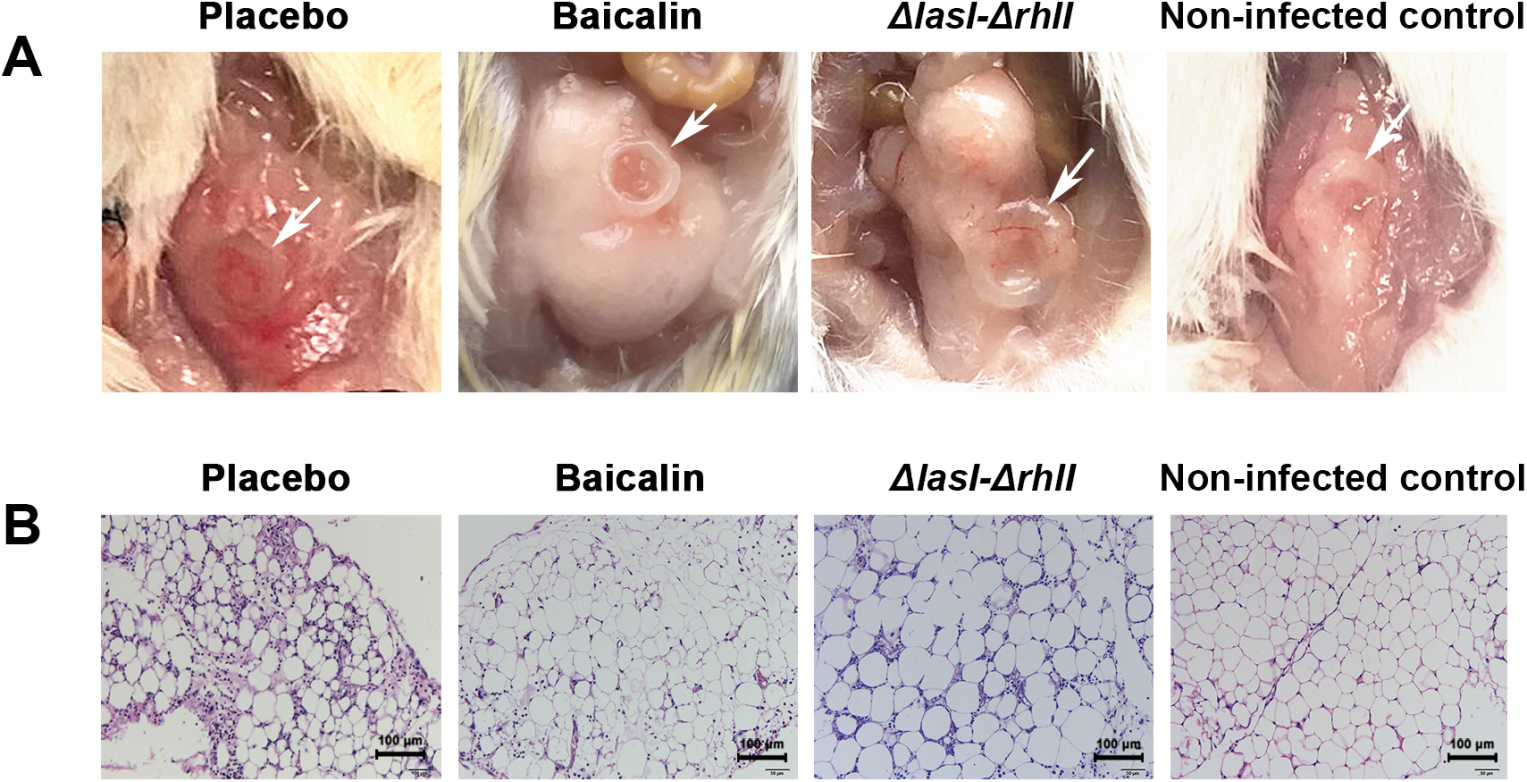
**Comparison of Gross (A) and Microscopic (B) Pathological Changes in the Peritoneal Tissue after Treatment with Vehicle or Baicalin.** Histopathology was performed using an upright microscope at 200× magnification. The white arrow indicates the implant.

To evaluate the impact of baicalin treatment on the pathological manifestations of peritoneal tissue injury surrounding the implants, we performed histopathologic analysis based on hematoxylin and eosin staining of lesion tissue at 3 days after peritoneal infection in mice that received either 100 mg/kg baicalin or PBS as a placebo control. Gross inspection revealed hyperemic and red peritoneal tissue around the *P*. *aeruginosa* PAO1-infected implants (untreated control group), whereas the *P*. *aeruginosa ΔlasI-ΔrhlI-*infected group exhibited a milder inflammatory appearance. Following treatment with baicalin, inflammation in the peritoneal tissue of *P*. *aeruginosa* PAO1-infected mice was markedly relieved, with a light pink appearance (Fig 11A). The histopathology of the untreated control group was dominated by significant neutrophil infiltration into the peritoneal tissue, accompanied by interstitial edema and capillary dilation. In contrast, baicalin treatment resulted in a marked reduction in inflammation, as indicated by the reduced accumulation of cellular infiltration in the peritoneal tissue. The peritoneal tissue of mice infected with *P*. *aeruginosa ΔlasI-ΔrhlI* also demonstrated milder inflammatory cell infiltration. As a negative control, the implantation of uninfected silicone tube rarely resulted in any apparent inflammation or alteration in gross and microscopic characteristics after 3 day’s insertion. (Fig 11B).

**Fig 12:**
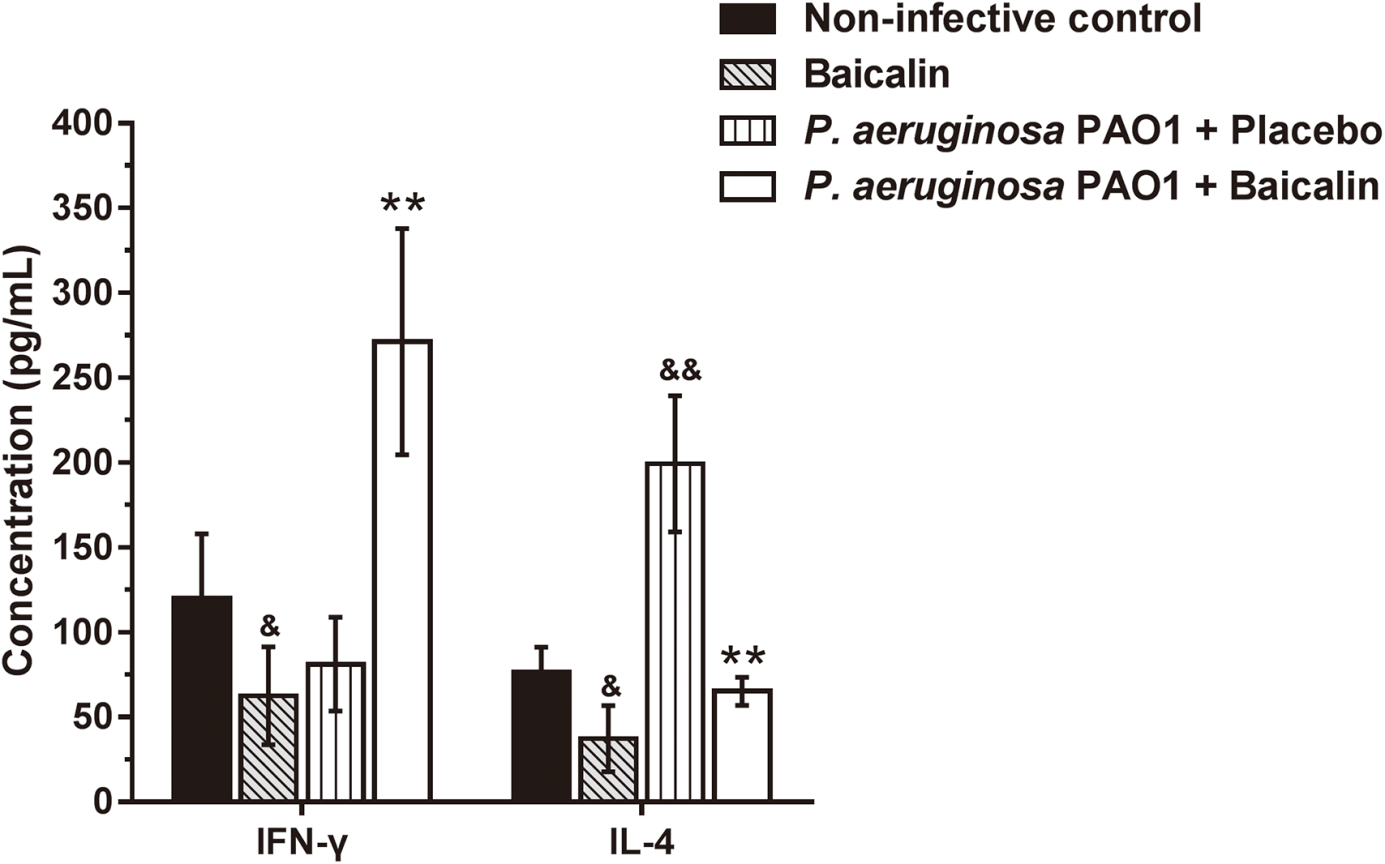
Cytokine levels of IFN-γ and IL-4 in fluid obtained by flushing the peritoneal cavities of mice treated with either baicalin or vehicle, measured by ELISA. The data show the average values of three independent experiments performed in duplicate. Values are the mean ± standard error. ^&^*P*<0.05 and ^&&^*P*<0.01 compared with the vehicle control group, **P<0.01 compared with the group in which the implant was pre-colonized with *P*. *aeruginosa* PAO1 but no baicalin was administered.

To clarify the mechanism by which baicalin alters *P*. *aeruginosa* PAO1 infection *in vivo*, we evaluated the levels of the cytokines IFN-γ and IL-4, which indicate activation of the Th1 and Th2 arms, respectively, in the fluid flushed from the mouse peritoneal cavity. Mice that received baicalin treatment showed a significant decrease in IL-4 in the peritoneal flushing fluid (*P*<0.01) compared with the untreated control group. In contrast, IFN-γ production was significantly increased after the infected mice were treated with baicalin compared with the untreated control group (*P*<0.01) (Fig 12). The IFN-γ/IL-4 ratio was 4.26±1.55 in the baicalin-treated group and 0.44±0.24 in the untreated group, indicating a reversal of the IFN-γ/IL-4 ratio upon baicalin treatment.

## Discussion

Plant-derived compounds have predominated in infection therapy for decades and are effective, low in toxicity and producing lower levels of acquired resistance [43]. Some compounds extracted from plants possess antibacterial activity with higher or overlapping MIC values and anti-QS and anti-biofilm activities even at lower concentrations [44,45]. Based on susceptibility testing, baicalin lacks anti-*P*. *aeruginosa* activity, with MICs exceeding 1024 μg/mL. Consequently, here, we used sub-MICs of baicalin (64, 128, and 256 μg/mL) that are insufficient to neutralize microbes, as monitoring the growth curves revealed no significant differences in growth patterns of *P*. *aeruginosa* when treated with these concentrations.

*P*. *aeruginosa* biofilm development occurs in two major steps: (1) cell–surface attachment, representing the initiation of biofilm formation via the adhesion of so-called “linking film” bacteria, which provide the groundwork for further biofilm growth; and (2) cell–cell interactions, which represent an accumulative phase in which the bacteria form microcolonies leading to the construction of a multilayer structure, culminating in biofilm development [7,46]. Zeng et al. [47] reported the ability of baicalin to inhibit *P*. *aeruginosa* attachment to a glass surface after 72 h of treatment at a concentration of 200 μM (nearly 89 μg/mL). However, no detailed analysis was carried out, particularly in terms of obtaining morphological evidence of the effects of this compound on biofilms. In the present study, we quantitatively and morphologically demonstrate striking dose- and time-dependent inhibition of *P*. *aeruginosa* biofilm formation by baicalin at concentrations of 64, 128, and 256 μg/mL. Based on these findings, baicalin inhibits the first major process by which *P*. *aeruginosa* cells attach to a surface and form the linking film, reduces the accumulation of microcolonies, and in turn hinders the development of intact and mature biofilms. Therefore, this property of baicalin shows promise for the prophylactic treatment of *P*. *aeruginosa* biofilm-associated infections.

Clinically, many biofilm-associated infections occur despite prevention under many circumstances. Once a biofilm has been established on an abiotic or tissue surface, eradication is nearly impossible with conventional doses of antibiotics [8]. Combinatorial treatment consisting of antibiotics and compounds exhibiting anti-biofilm activity is considered one potential strategy for solving this problem. For instance, Mu et al. [48] revealed that chitosan enhanced the bactericidal efficacy of gentamycin against *Listeria* species in biofilms by facilitating the entry of antibiotics into the biofilm architecture. Chen et al. [49] demonstrated the ability of baicalein, a derivative of baicalin, synergized with vancomycin to induce remarkable dispersive effects on *S*. *aureus* biofilms by eradicating extracellular matrices and eliminating sessile *S*. *aureus* cells from within the biofilms. To determine whether baicalin and antibiotics exert synergistic effects on the dispersal of pre-existing *P*. *aeruginosa* biofilms, this study used three classes of antibiotics representing different antibiotic families that are widely used in the clinic to treat *P*. *aeruginosa* infection. These antibiotics were levofloxacin (belonging to the fluoroquinolone family), amikacin (belonging to the aminoglycoside family) and ceftazidime (belonging to the cephalosporin family). Our quantitative assay results and morphological observations (CLSM, SEM) indicated that a single treatment with baicalin at sub-MICs or with tested antibiotics elicited a mild dispersal effect on the *P*. *aeruginosa* biofilm biomass and was ineffective to kill live bacteria in biofilms compared with a control, primarily due to the lack of a direct bactericidal effect of baicalin at sub-MICs and the inability of antibiotics at MBCs alone to penetrate the biofilm architecture and to kill highly resistant biofilm cells. Interestingly, when *P*. *aeruginosa* biofilms were exposed to a mixture of baicalin and individual antibiotics, fewer viable cells and biomass remained compared with the baicalin or antibiotics alone. This observation may be considered an extension of the research of Brackman et al. [16], as our observations indicated the ability of baicalin co-treatment with three different antibiotics to enhance the antimicrobial activity of those antibiotics, not only that of tobramycin. Our results also validate the effectiveness of baicalin/antibiotic combinations in eliminating both 24-h and 96-h *P*. *aeruginosa* biofilms, and this effect on biofilms becomes more marked with increasing concentrations of baicalin. The regulatory action of QS is known to be involved throughtout the process of *P*. *aeruginosa* biofilm formation, development and maturation. Thus, baicalin likely penetrated the *P*. *aeruginosa* biofilms, interfered with cell signaling, and repressed QS in the majority of biofilm-associated cells.

Traditional Chinese medicines harbor numerous antibacterial components with different mechanisms. Some of these compounds exert QS inhibitory activity against *P*. *aeruginosa*, such as sodium houttuyfonate, tea polyphenols, andrographolide and baicalein, an aglycone derivative of baicalin [44,45,50,51]. In this work, we investigated the potential ability of baicalin at sub-MICs to inhibit QS genes, signaling molecules and associated virulence factors in pathogenic *P*. *aeruginosa* PAO1 and assessed whether baicalin possesses anti-QS activity. Secreted extracellular virulence factors are indicators of the optimal function of the QS regulon in *P*. *aeruginosa* [17,18,52]. Their reduced production supports the anti-QS potential of the tested compound. LasA protease and LasB elastase play important roles in the pathogenesis of *P*. *aeruginosa*-induced respiratory tract infections by assisting with colonization and rupturing the host tissue [17]. Pyocyanin, a phenazine derivative, generates reactive oxygen species by oxidizing reduced glutathione in cells and simultaneously reducing oxygen [53]. Rhamnolipids serve as important surfactants that facilitate *P*. *aeruginosa* surface motility, which is required for biofilm initiation [54]. Exotoxin A is the most toxic substance produced by *P*. *aeruginosa* and inhibits intracellular protein synthesis via the same mechanism as utilized by diphtheria toxin [36]. In the present study, baicalin reduced the production of the majority of these virulence factors at increasing sub-MICs of 64 and 256 μg/mL, although the reduction induced by the lower concentration of baicalin (64 μg/mL) against exotoxin A was not statistically significant. The higher dose of baicalin (256 μg/mL) clearly reduced bacterial numbers to levels comparable to those of *ΔlasI-ΔrhlI*, supporting the ability of baicalin to attenuate QS-related virulence factors in *P*. *aeruginosa*. Notably, exogenous supplementation with corresponding HSLs may potentially relieve the inhibitory effects of baicalin on the secretion of extracellular virulence factors, indicating that the attenuation of *P*. *aeruginosa* virulence by baicalin may be partially attributable to the reduced synthesis of HSLs, which are critical signaling molecules that activate the QS circuit and then produce extracellular virulence factors.

The processes of biofilm formation and development are invariably initiated by attachment and colonization, which is crucially mediated by flagellar motilities (swimming and swarming) and, during later stages, by twitching motility [55]. Unlike the former two motilities, twitching motility is mediated by the successive extension and retraction of polar Type IV pili [56]. *P*. *aeruginosa* motility is positively regulated by both QS signals, *las* and *rhl*. QS-deficient strains lacking motility consistently form dispersed and thin biofilms, and disabling any QS component affects bacterial motility [57]. Based on our results, baicalin at sub-MICs did not reduce the swimming ability of *P*. *aeruginosa* but markedly affected both swarming and twitching motility, indicating the ability of baicalin to interfere with the functions of flagella and Type IV pili. Since motilities are regulated by QS, the inhibitory effects of baicalin on *P*. *aeruginosa* motilities may occur either by interfering with the QS circuit or by directly acting on flagella and Type IV pili, thereby contributing to the inhibition of bacterial adhesion and colonization, biofilm formation and the production of other QS-controlled virulence factors.

The anti-QS activity of baicalin also appears to inhibit normal QS circuitry in *P*. *aeruginosa* PAO1 via molecular and genetic mechanisms. At a high cell density, a high concentration of AHLs leads to the incorporation of *lasR* into the AHL-lasR complex, which serves as a transcriptional activator and triggers the expression of downstream target genes within the QS regulon, such as *lasI*, *lasR*, *rhlI*, and *rhlR* [58]. Real-time PCR results showing downregulated expression of these genes supported our speculation regarding the anti-virulence activity of baicalin. The reduced expression of these genes may not only lower the production of several QS-controlled virulence factors but also interfere with the generation of QS signaling molecules and biofilm formation. In addition, the quinolone signal is linked to fluoroquinolone resistance, affecting biofilm formation and participating in the transcription of *rhl*-controlled virulence genes in *P*. *aeruginosa* [59]. Reduced expression of the *pqsA* and *pqsR* genes after treatment with baicalin was also demonstrated in this study, thereby elucidating the mechanism underlying the synergism of baicalin with LEV to eradicate *P*. *aeruginosa* biofilms as demonstrated above. Additionally, the real-time PCR results were supported by HPLC-MS data showing reductions in the peak intensities of 3-oxo-C_12_-HSL and C_4_-HSL after baicalin exposure. These results are consistent with a previous study reported by our group [28], in which baicalein, the aglycone derivative of baicalin, was shown to exert anti-QS activity in *P*. *aeruginosa* PAO1 by inhibiting the expression of QS genes and inhibiting AHL production. As baicalin and baicalein constitute the major active constituents of *Scutellaria baicalensis*, the discovery of their QS inhibitory potential will actually improve the medicinal value of *Scutellaria baicalensis* in the field of anti-biofilm infection.

Taken together, baicalin at sub-MICs exerts global inhibitory effects on the *P*. *aeruginosa* QS circuitry, thus affecting the *in vitro* production of QS-controlled virulence factors, motility and biofilm formation. Increased sensitivity to antibiotics depends on the process of QS, with the baicalin-induced reduced thickness of *P*. *aeruginosa* biofilms eventually leading to increased susceptibility of *P*. *aeruginosa* PAO1 biofilms to LEV, AMK and CAZ. Additionally, baicalin treatment may also be associated with the inhibition of QS-controlled virulence factors, which are important for maintaining biofilm architecture and maturation resistance to antibiotics [12,17].

The susceptibility of *C*. *elegans* to various virulence phenotypes of *P*. *aeruginosa* makes this invertebrate nematode an appropriate model for studying host–pathogen interactions [60]. Thus, we further investigated the effects of baicalin on the pathogenesis of *P*. *aeruginosa* regulated by QS using a *C*. *elegans* infectivity model. A considerable increase in the survival rate of *C*. *elegans* was observed following sub-MIC baicalin treatment compared with the non-baicalin control group. Moreover, CFU-counting analyses of the guts of worms fed on baicalin-treated and untreated *P*. *aeruginosa* PAO1 revealed similar numbers of bacteria recovered from nematodes. Because bacterial proliferation in the nematode gut is directly proportional to worm death, we speculated that baicalin at sub-MICs was able to attenuate the pathogenesis of *P*. *aeruginosa* PAO1 by suppressing the production of QS-controlled virulence factors rather than by exerting direct bactericidal effects. According to a previous study, pyocyanin, one of the QS-controlled virulence factors produced by *P*. *aeruginosa*, causes the lethal paralysis of muscular tissue in *C*. *elegans*, leading to asphyxia and worm death within 24 h [61]. Additionally, *P*. *aeruginosa* motility is responsible for colonization and dissemination in the worm gut [62]. Baicalin inhibited motility and pyocyanin production, and these effects may underlie the increase in the LT_50_ of *C*. *elegans* fed on baicalin-treated *P*. *aeruginosa*. In addition, the *P*. *aeruginosa* QS mutant characterized by reduced virulence contributed to the longer survival of *C*. *elegans* compared with wild-type *P*. *aeruginosa* PAO1, further supporting our speculation.

*P*. *aeruginosa* infections related to the use of indwelling catheters and foreign-body implants are serious complications of medical device insertion in the clinic [6,63]. Therefore, we extended from our previous results in a mouse peritoneal implant infection model. Previous studies demonstrated the ability of functional QS systems to play vital roles in the persistence of *P*. *aeruginosa* in a pulmonary infection model and during foreign-body infection [15,64]. Treatment with compounds possessing QS inhibitory activities, such as furanone C-30, horseradish juice extract and ajoene [20], reduces bacterial counts in both *in vivo* mouse models of infection. In the present study, compared with the control group, initiating treatment with baicalin significantly reduced the number of CFUs associated with implants after *P*. *aeruginosa* biofilm infection for 3 days. Furthermore, consistent with our previous *in vitro* results, significantly fewer bacteria were recovered from the implants after combinatorial treatment with baicalin and antibiotics, specifically LEV, AMK and CAZ, compared with the control group and single-treatment groups. As our pilot pharmacokinetics study showed that the dose of baicalin used in this study led to a serum *Cmax* in mice within the range in which baicalin effectively acted on QS *in vitro*, we speculate that baicalin serves as a QS inhibitor, facilitates the ability of mice to clear a foreign-body infection and enhances the bactericidal efficacy of conventional antibiotics *in vivo*.

Biofilm infections are characterized by persistent local inflammation [65]. The production of QS-controlled virulence factors strongly induces an inflammatory response and tissue damage. For example, pyocyanin, a redox-active secondary metabolite, mediates persistent tissue damage and necrosis during *P*. *aeruginosa* infection [66]. Protease and elastase disrupt the interstitial tissue and lead to the expansion of infection foci [53]. Rhamnolipids function as protective shields against the innate immune system; such contact results in the necrosis of polymorphonuclear leukocytes [67]. In contrast, the *P*. *aeruginosa* QS gene mutant has weaker pathogenicity and a diminished ability to cause tissue damage, as demonstrated in the present study. Interestingly, baicalin alleviated the inflammatory response and mitigated tissue damage during *P*. *aeruginosa* infection; this may result from baicalin quenching the QS circuitry *in vivo*, leading to inhibition of the production of QS-controlled virulence factors and, ultimately, underscoring its anti-*P*. *aeruginosa* biofilm infection activity. According to the recently published ESCMID guidelines for biofilm infection, the predominant immune response to the microbes present in *P*. *aeruginosa* biofilms exhibits either Th2 (antibody) or Th1 (cell-mediated) polarization [68], distinguished by the activation of IL-4 and IFN-γ, respectively. The Th2 immune response frequently plays a dominant role during persistent infections caused by *P*. *aeruginosa* biofilms. However, a shift from the Th2 to the Th1 immune response may promote bacterial clearance and improve the prognosis of infected individuals [69,70]. According to Ma et al., baicalin treatment effectively reduced IL-4 levels and increased IFN-γ levels in the bronchoalveolar lavage fluid in a mouse model of allergic asthma [71]. Likewise, based on the results of the present study, baicalin significantly reduced the production of IL-4 and enhanced the generation of IFN-γ in the lavage fluid collected from the peritoneal cavity, reversing the Th1/Th2 balance to a Th1-dominated state. Thus, modifying the type of immune response raised against *P*. *aeruginosa* biofilms via baicalin treatment may facilitate pathogen elimination by the immune system, resolve inflammation and improve the prognosis of implant-related infection.

In summary, the present study highlights the ability of baicalin to effectively inhibit *P*. *aeruginosa* biofilm formation, enhance the permeability and bactericidal effects of conventional antibiotics against biofilm cells and attenuate virulence by interfering with the QS circuit of *P*. *aeruginosa in vitro*. The *in vivo* results obtained both in *C*. *elegans* and in a mouse model of peritoneal foreign-body infection correlated well with the *in vitro* findings. Additionally, the infected mice benefited from the immune-modifying activity of baicalin, which was associated with a good prognosis. The US FDA has strongly encouraged monitoring the active ingredients, representative markers and/or major chemical components of TCM and TCM formulas [23]. As a monomeric component of *Scutellaria baicalensis* with a well-defined chemical structure (S3 Fig), baicalin has been made into variants of TCM formulae for adjuvant treatment of clinical infection, such as the Qingkailing injection, in which this component was believed to be safe for clinical use [23]. Thus, baicalin is considered to have potential for selection as a novel anti-biofilm and anti-QS compound against *P*. *aeruginosa*, particularly for implant-related infections. Nevertheless, further studies are required to clarify the details of the pharmacokinetic, pharmacodynamic and toxicologic characteristic of baicalin monotherapy on animals infected with *P*. *aeruginosa* biofilm before baicalin enters clinical trials.

## Supporting information

S1 Fig Effects of baicalin on the dispersion of *P*. *aeruginosa* PAO1 biofilms. Twenty-four-hour *P*. *aeruginosa* PAO1 biofilms were exposed to sub-MICs (64, 128, and 256 μg/mL) of baicalin for 24 h. The remaining biofilm mass (A) and bacterial counts (B) were quantified. The data show the average value of three independent experiments performed in duplicate. Values are shown as the mean ± standard error. *P<0.05 compared with the control group.
(DOCX)

S2 Fig The serum concentrations of baicalin in non-infective BALB/c mice after Subcutaneous administration of a single bolus dose (25, 50, or 100 mg/kg) of baicalin. (DOCX)

S3 Fig Chemical structure of baicalin. Molecular weight: 446.37 g/mol.
(DOCX)

S1 Table Precursor ions [M+H]^+^ and fragmentation ions derived from the acyl chain moiety [M+H-101]^+^ of AHLs were detected and identified by HPLC-MS. (DOCX)

S2 Table Susceptibility test results for antimicrobial agents against the quality control strain *P*. *aeruginosa* ATCC27853. (DOCX)

S3 Table Primers utilized for real-time PCR. (DOCX)
