# Supplementary figures and images for: Baicalin inhibits biofilm formation, attenuates the quorum sensing-controlled virulence and enhances Pseudomonas aeruginosa clearance in a mouse peritoneal implant infection model
Source: PLoS One. 2017 Apr 28;12(4):e0176883. doi: 10.1371/journal.pone.0176883 (PMC5409170; doi:10.1371/journal.pone.0176883)

**S2 Fig**


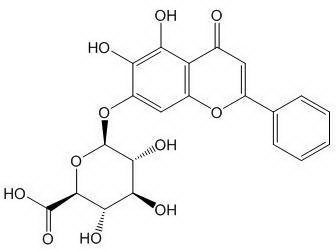

Supplement: S2 Fig — (DOCX) [file pone.0176883.s002.docx]
