# Supplementary material for: Baicalin inhibits biofilm formation, attenuates the quorum sensing-controlled virulence and enhances Pseudomonas aeruginosa clearance in a mouse peritoneal implant infection model
Source: PLoS One. 2017 Apr 28;12(4):e0176883. doi: 10.1371/journal.pone.0176883 (PMC5409170; doi:10.1371/journal.pone.0176883)
Supplement: S3 Fig — Molecular weight: 446.37 g/mol. (DOCX) [file pone.0176883.s003.docx]

**S3 Fig**





**Methods for pharmacokinetic determination**

The pharmacokinetics of baicalin in non-infected BALB/c mice after subcutaneous administration of a single bolus dose (25, 50, or 100 mg/kg) of baicalin was determined prior to the combination treatment study on foreign-body implant infection mice. Briefly, healthy female BALB/c mice aged 8 weeks and weighing 18-22 g were divided into fourteen groups, each group corresponding to a single time point and containing five mice. Baicalin prepared as described in the material and methods section was injected subcutaneously in a volume of 100 µL. At various timepoints after administration (0, 0.25, 0.5, 1, 2, 3, 4, 6, 8, 10, 12,16, 20 and 24 h), mice were euthanized. Then, blood was aseptically sampled from the heart with a 1 ml syringe and transferred to a sterile 1.5-mL Eppendorf tube. After centrifugation at 12,000 rpm at 4°C, the serum was retained in a centrifuge tube (1.5 mL) and stored at -20°C until determination of the serum concentration using HPLC (see reference published at “http://d.g.wanfangdata.com.cn/Periodical_zcy200506031.aspx” in Chinese). Chromatographic conditions: A Shim-pack ODS column (5 µm, 4.6 mm×250 mm) was kept at 37°C. The gradient elution program using acetonitrile with 0.1% formic acid (A) and acetonitrile (B) as the mobile phase was as follows: 0-1 min, 10%–55% A; and 1-10 min, 55% A. The flow rate was set at 1.5 mL/min, and the injection volume was 20 µL. For preparing HPLC samples, 100 μL of collected serum was mixed with 50 μL of 1 mmol/L KH_2_PO_4_ and then supplemented with 100 μL of acetonitrile-methanol (1:l, v/v). After 10 minutes of vortexing and centrifugation at 12,000 rpm for 10 minutes, the supernatant was collected, and 20 µL was directly injected onto the HPLC system for analysis. Pharmacokinetic parameters, including Cmax, *AUC* (0-∞) and MRT (0-∞), were calculated using Drug and Statistics Software (DAS, Mathematical Pharmacology Professional Committee of China, Shanghai, China).
