# Supplementary material for: Baicalin inhibits biofilm formation, attenuates the quorum sensing-controlled virulence and enhances Pseudomonas aeruginosa clearance in a mouse peritoneal implant infection model
Source: PLoS One. 2017 Apr 28;12(4):e0176883. doi: 10.1371/journal.pone.0176883 (PMC5409170; doi:10.1371/journal.pone.0176883)
Supplement: S1 Table — (DOCX) [file pone.0176883.s004.docx]

**S1 Table. Precursor Ions [M+H]^+^ and Fragmentation Ions Derived from the Acyl Chain Moiety [M+H-101]^+^ of AHLs Were Detected and Identified by HPLC-MS.**

| AHLs | Retention time (min) | [M+H]+ ion (m/z) | Fragmentation ions (m/z) |
| --- | --- | --- | --- |
| 3-oxo-C12-HSL | 12.44 | 298 | 197 |
| C4-HSL | 3.02 | 172 | 71 |
