# Supplementary material for: Baicalin inhibits biofilm formation, attenuates the quorum sensing-controlled virulence and enhances Pseudomonas aeruginosa clearance in a mouse peritoneal implant infection model
Source: PLoS One. 2017 Apr 28;12(4):e0176883. doi: 10.1371/journal.pone.0176883 (PMC5409170; doi:10.1371/journal.pone.0176883)
Supplement: S2 Table — (DOCX) [file pone.0176883.s005.docx]

**S2 Table. Susceptibility Test Results for Antimicrobial Agents Against the Quality Control Strain *P. aeruginosa* ATCC27853.**

| Antimicrobial agents | MIC | CLSI |
| --- | --- | --- |
| Baicalin (μg/mL) | ＞1024 | - |
| Levofloxacin (μg/mL) | 0.5 | 0.5-4 |
| Amikacin (μg/mL) | 1 | 1-4 |
| Ceftazidime (μg/mL) | 1 | 1-4 |
