# Supplementary material for: Baicalin inhibits biofilm formation, attenuates the quorum sensing-controlled virulence and enhances Pseudomonas aeruginosa clearance in a mouse peritoneal implant infection model
Source: PLoS One. 2017 Apr 28;12(4):e0176883. doi: 10.1371/journal.pone.0176883 (PMC5409170; doi:10.1371/journal.pone.0176883)
Supplement: S3 Table — (DOCX) [file pone.0176883.s006.docx]

**S3 Table. Primers Utilized for Real-time PCR.**

| Target gene | Type | Primer sequences |
| --- | --- | --- |
| 16S rRNA (PA 5369.5) | Fw | 5’-CGTCCGGAAACGGCCGCT-3’ |
|  | Rev | 5’-CTCTCAGACCAGTTACGG-3’ |
| *lasI* (PA 1432) | Fw | 5’-AGGCGTGGAGAAGATGATG-3’ |
|  | Rev | 5’-ATCTGGGTCTTGGCATTGAG-3’ |
| *lasR* (PA 1430) | Fw | 5’-GTGGAAAATTGGAGTGGAGCG-3’ |
|  | Rev | 5’-GTAGTTGCCGACGATGAAGG-3’ |
| *rhlI* (PA 3476) | Fw | 5’-TGCAGCCATTCCGGGTGGTA-3’ |
|  | Rev | 5’-TCGTTCGCAACGGCGTGAT-3’ |
| *rhlR* (PA 3477) | Fw | 5’-GGGTTGGACATCAGCATCGG-3’ |
|  | Rev | 5’-TTGCTCAGCGTGCTTTCCGT-3’ |
| *PqsA* (PA 0996) | Fw | 5’-GACCGGCTGTATTCGATTC-3’ |
|  | Rev | 5’-GCTGAACCAGGGAAAGAAC-3’ |
| *PqsR* (PA 0964) | Fw | 5’-CTGATCTGCCGGTAATTGG-3’ |
|  | Rev | 5’-ATCGACGAGGAACTGAAGA-3’ |
